# Extrapolating empirical long-term survival data: the impact of updated follow-up data and parametric extrapolation methods on survival estimates in multiple myeloma

**DOI:** 10.1186/s12874-023-01952-2

**Published:** 2023-05-29

**Authors:** LJ Bakker, FW Thielen, WK Redekop, CA Uyl-de Groot, HM Blommestein

**Affiliations:** 1 Erasmus School of Health Policy and Management, Erasmus University Rotterdam, P.O. Box 1738, Rotterdam, 3000 DR The Netherlands; 2 Erasmus Centre for Health Economics Rotterdam, Erasmus University, Rotterdam, The Netherlands

**Keywords:** Survival, Parametric Extrapolation, Multiple myeloma, Kaplan-Meier

## Abstract

**Background:**

In economic evaluations, survival is often extrapolated to smooth out the Kaplan-Meier estimate and because the available data (e.g., from randomized controlled trials) are often right censored. Validation of the accuracy of extrapolated results can depend on the length of follow-up and the assumptions made about the survival hazard. Here, we analyze the accuracy of different extrapolation techniques while varying the data cut-off to estimate long-term survival in newly diagnosed multiple myeloma (MM) patients.

**Methods:**

Empirical data were available from a randomized controlled trial and a registry for MM patients treated with melphalan + prednisone, thalidomide, and bortezomib- based regimens. Standard parametric and spline models were fitted while artificially reducing follow-up by introducing database locks. The maximum follow-up for these locks varied from 3 to 13 years. Extrapolated (conditional) restricted mean survival time (RMST) was compared to the Kaplan-Meier RMST and models were selected according to statistical tests, and visual fit.

**Results:**

For all treatments, the RMST error decreased when follow-up and the absolute number of events increased, and censoring decreased. The decline in RMST error was highest when maximum follow-up exceeded six years. However, even when censoring is low there can still be considerable deviations in the extrapolated RMST conditional on survival until extrapolation when compared to the KM-estimate.

**Conclusions:**

We demonstrate that both standard parametric and spline models could be worthy candidates when extrapolating survival for the populations examined. Nevertheless, researchers and decision makers should be wary of uncertainty in results even when censoring has decreased, and the number of events has increased.

**Supplementary Information:**

The online version contains supplementary material available at 10.1186/s12874-023-01952-2.

## Introduction

The data available for assessing efficacy of novel healthcare technologies in oncology often comes from randomized controlled trials (RCTs). However, RCTs do not provide all necessary information for assessing the *cost-effectiveness* of these technologies. RCTs often have limited follow-up times and thus increased censoring at market approval while a lifetime horizon is usually recommended in best-practice guidelines for economic evaluations [1, 2]. This lifetime horizon ensures that all differences (i.e., short- and long-term) of the technologies compared are accounted for. Since a lifetime horizon almost always exceeds the follow-up duration of RCTs or other data sources used in economic evaluations (e.g., registries), empirical survival data are typically right censored [3]. For the novel treatment assessed, this can result in considerable uncertainty regarding the parametric survival function. For the comparator, this depends on whether the treatment administered in the trial is representative of what happens in current care and whether alternative sources of data are available to inform long-term survival.

There is substantial variation in the percentage of patients that is right censored depending on the type of disease [4]. For hematological malignancies for instance, the average percentage censored was 84% in initial publications and 54% in updated publications, whereas for other malignancies this varied from 28 to 73% in the initial publication and 13-47% in updated results [4]. With an increase in novel immunotherapies resulting in prolonged survival for multiple myeloma patients, such as daratumumab and lenalidomide [5, 6], this issue has become even more prominent in recent years.

To address the issue of right-censoring, parametric survival functions and other methods for extrapolation are used to estimate long-term survival, making assumptions about the underlying hazard function for the extrapolated period based on the data observed [7]. Many types of models can be used to extrapolate survival from empirical evidence. Standard parametric models are generally included (e.g., Weibull, lognormal), but it is recommended to also consider more flexible models (e.g., spline, parametric mixture models) that allow for multiple turning points in the hazard function [8]. More flexible parametric spline models for instance were found in a previous study by Gray et al. to predict 10-year survival quite accurately for large cohorts of registry patients for which there was little uncertainty in the data [9].

Assessing the suitability of models and selecting the best-fitting model for extrapolation can be done through inspection of log cumulative hazard plots, inspection of visual fit, and statistical tests (e.g., Akaike Information Criterion (AIC) or Bayesian Information Criterion (BIC)) [10]. Real-world data may also guide model selection by assessing whether extrapolated results are plausible when compared to patient survival outside the context of a clinical trial [11]. Prior research has suggested that model selection should consider the length of follow-up of the data available [12]. In a case study, Bullement et al. assessed the accuracy of extrapolations for four different data-cuts of the JAVELIN Merkel 200 trial which studied the treatment effect of avelumab for patients with Merkel cell carcinoma. The authors found that extrapolations using longer follow-up (e.g., 36 months) favored more flexible spline-based models [12].

Despite this guidance, selecting a good fitting model and analyzing the uncertainty surrounding model choice remains a challenging endeavor and several publications already assessed the accuracy of extrapolations (e.g., [4, 7–9, 11–18]). These studies vary in the type of disease treated (e.g., melanoma, lung cancer), type of treatment evaluated (e.g., immunotherapy, surgery), the type of models compared, the duration of empirical follow-up time, the availability of individual patient data (IPD) and recreated data from published RCTs, their sample size, as well as the inclusion of external data sources. The overall accuracy of extrapolations has been found to be correlated with the percentage censored [4]. Everest et al. conducted a systematic review to find published RCTs with initial and updated results. For the 32 eligible RCTs, the accuracy of extrapolations based on the initial publication was then assessed after reconstructing individual patient data and fitting standard parametric models. The authors found that the difference between the extrapolated survival and the empirical survival increased as the percentage of patients censored increased [4].

In this study, we aim to compare extrapolation methods to assess the relationship between data maturity and survival projection accuracy, in the presence of several data sources. Both standard parametric models and spline models were fitted to RCT and patient registry data from patients with multiple myeloma while varying the maximum data cut-off (DCO) times. These extrapolations were not informed by alternative sources of information assuming that solely the dataset at hand with its particular DCO would be the best source available for extrapolation. The resulting extrapolations were compared to long-term empirical survival to determine the best candidate models. The results of our study may assist researchers in assessing whether the IPD is sufficiently mature for cost-effectiveness analysis and guide their decision-making concerning the sensitivity analyses that should be conducted.

## Methods

### Patient population & data

All details on the data sources, treatment arms, inclusion dates, and the data cuts can be found in Table 1. IPD from an RCT performed by the Dutch Haemato-oncology Foundation for Adults in the Netherlands (HOVON), and data from the Dutch National Cancer Registry (NKR) were used to assess the accuracy of extrapolations compared to long-term empirical survival. The HOVON49 study compared melphalan + prednisone (HOVON - MP) with melphalan + prednisone + thalidomide (HOVON - Thal) in newly diagnosed multiple myeloma patients > 65 years of age [19]. Patients were included between September 2002 and July 2007 and long-term follow-up was available until December 2015.

**Table 1:** Cohorts used to compare long term extrapolations for newly diagnosed multiple myeloma including the number of patients included and data cuts

Data Source	Drug	Mean age of cohort	Years of inclusion	Start date Inclusion	End date Inclusion	Sample Size	Data cuts	Maximum Follow-up (years)	Minimum Potential Follow-up (years)	Horizon RMST (years)	Horizon lifetime RMST (years)
HOVON49	MP	74	2002–2007	09-19-2002	07-16-2007	168	2005, 2008, 2010, 2012	3, 6, 8, 10	n.a.,1,3,5	11	35
HOVON49	Thal.	74	2002–2007	09-19-2002	06-27-2007	166	2005, 2008, 2010, 2012	3, 6, 8, 10	n.a.,1,3,5	11	35
PHAROS	MP	76	2004–2009	01-15-2004	01-15-2009	208	2007, 2010, 2012, 2014, 2017	3, 6, 8, 10,13	n.a.,1,3,5,8	14	35
PHAROS	Thal.	72	2005–2010	01-01-2005	01-01-2010	552	2008, 2011, 2013, 2015, 2018	3, 6, 8, 10, 13	n.a.,1,3,5,8	14	35
PHAROS	Bort.	64	2006–2011	01-01-2006	01-01-2011	122	2009, 2012,2014, 2016, 2019	3, 6, 8, 10, 13	n.a.,1,3,5,8	14	35
NKR+	Bort.	74	2014–2016	01-10-2014	01-10-2016	637	2017, 2020, 2022	3, 6, 8	1,4,6	8	35

Bort = Bortezomib based, HOVON = Dutch Haemato-oncology Foundation for Adults in the Netherlands, MP = melphalan + prednisone, NKR + = Dutch National Cancer Registry, PHAROS = Population based HAematological Registry for Observational Studies, RMST = Restricted mean survival time, Thal.= Thalidomide based

Data from the NKR registry, including the Dutch Population based HAematological Registry for Observational Studies (PHAROS), were also used [20, 21]. From the PHAROS database, newly diagnosed patients that received first-line treatment with MP (PHAROS - MP), thalidomide (PHAROS - Thal) and bortezomib (PHAROS - Bort) based regimens were included. Patients receiving melphalan + prednisone + bortezomib (NKR+ - MPV) present in the NKR + data were also included as a separate cohort. The mean age of the PHAROS-Bort cohort was slightly lower (Table 1) because at the time bortezomib was not the recommended first-line treatment for all multiple myeloma patients. In 2006–2011, bortezomib based regimens were mainly prescribed in younger patients followed by patients with kidney failure [22]. All dates for inclusion and exclusion can be found in Table 1 and Figure S1. For all patients in the PHAROS database and the NKR + database, follow-up was included up until January 2022.

Overall survival was extrapolated using data sets which varied in the maximum follow-up time after the start of patient inclusion. For the MP arm from the HOVON49 study for instance, four datasets were created. In the first, patients were included from September 2002 until September 2005. Thus, the maximum follow-up of patients was three years and only patients that were enrolled before September 2005 were included. For the second HOVON - MP dataset, all enrolled patients were included (since enrollment ended in July 2007) but the final follow-up date was six years after starting enrollment (i.e., September 2008) and so forth.

The DCOs (i.e., < 3, 6, 8, 10, and 13 years) were chosen based on previously reported results from Everest et al. and Bullement et al. [4, 12] and according to the maximum potential follow-up in the dataset. For instance, if inclusion started in 2002 and the DCO was 2005 the longest that a patient could have been followed was 3 years (Table 1). The minimum amount of potential follow-up time for all patients included in the data is also reported in Table 1. For instance, when the maximum follow-up was < 6 years for the HOVON-MP arm the minimum potential follow-up for all patients included was 1 year and when the maximum follow-up was < 8 years the minimum potential follow-up was 3 years.

### Fitted models

The models used to extrapolate results included all commonly used standard parametric models recommended by the Technical Support Documents 14 and 21 from the National Institute for Health and Care Excellence Decision Support Unit (i.e., exponential, Weibull, Gompertz, Gamma, log-logistic, lognormal, Generalized Gamma) and spline models. Spline-based models are flexible models where the survival function is transformed by a link function using natural cubic splines [23]. Natural cubic splines impose monotonicity for the tails where the number at risk is low, whereas at earlier points monotonicity is guaranteed due to the data density if the sample size is reasonable [23]. The transformed survival function is then smoothed reducing the risk of sudden deviations especially in the tail. Knots are placed at extreme values of the survival times and internally [23]. Here, the number of knots was varied from one to three and a hazard, odds, and normal scale were used.

### Model selection & accuracy of predictions

In the results, we presented the models that had the lowest AIC, lowest BIC, and the best visual fit based on survival, and hazard plots. To select those models with the best visual fit, two authors (LB, HB) reviewed all curves independently. For the best visual fit, curves were selected based on four criteria: their fit to the Kaplan-Meier survival curve, the feasibility of the extrapolated survival, their fit to the smoothed hazard and the feasibility of the extrapolated hazard. If, based on these four criteria, multiple models were still eligible for ‘best’ fit, the model with the smallest number of parameters that needed to be estimated was selected. For instance, an exponential distribution for which one parameter needs to be estimated would be preferred over a generalized gamma distribution (three parameters). After individual selection, any remaining discrepancies were resolved by discussion to reach consensus. A third author (FT) participated in these discussions to resolve any ties in model selection. In preparation of the discussion the third author randomly assessed one-third of all curves according to the criteria noted above.

The accuracy of predictions was estimated using the restricted mean survival time (RMST). The RMST is equal to the mean survival restricted to a maximum time *t* instead of lifetime. It can be calculated by estimating the area under the curve (AUC) up until time *t* using integration [24]. All models were fitted and RMST estimated using the flexsurv package (version 2.1) in R [25]. First, a lifetime RMST was estimated for all cohorts. Here, the AUC was estimated for the extrapolated survival curves with the time horizon set to 35 years. Hereafter, the extrapolated survival was compared to the empirical survival with the horizon for RMST depending on the length of follow-up in empirical survival (Table 1). The RMST error was defined as the difference between the RMST from extrapolated curves and the RMST for the KM-estimate. In the second set of analyses, RMST was limited to the extrapolated proportion of the survival curve. Here, RMST was estimated conditional on surviving up until the point from where on extrapolation was required. Thus, for the data set with a maximum of three years follow-up, RMST was estimated conditional on having survived 3 years. Variations in RMST error were also plotted according to the percentage censored, absolute number of events, and the type of model (i.e., standard parametric or spline). For the spline models, knots were automatically placed at the centiles following recommendations by Royston & Parmar, when using the flexsurv package [23]. R version 4.0.3 was used for all analyses together with the packages flexsurv, muhaz, survRM2, lme4.

### Ethical approval

Approval for use of the PHAROS and NKR + data was granted through the supervisory committee of the Dutch Integral Cancer Registry. Approval for secondary use of the data from the HOVON49 study was provided by HOVON.

## Results

**Fig. 1:**
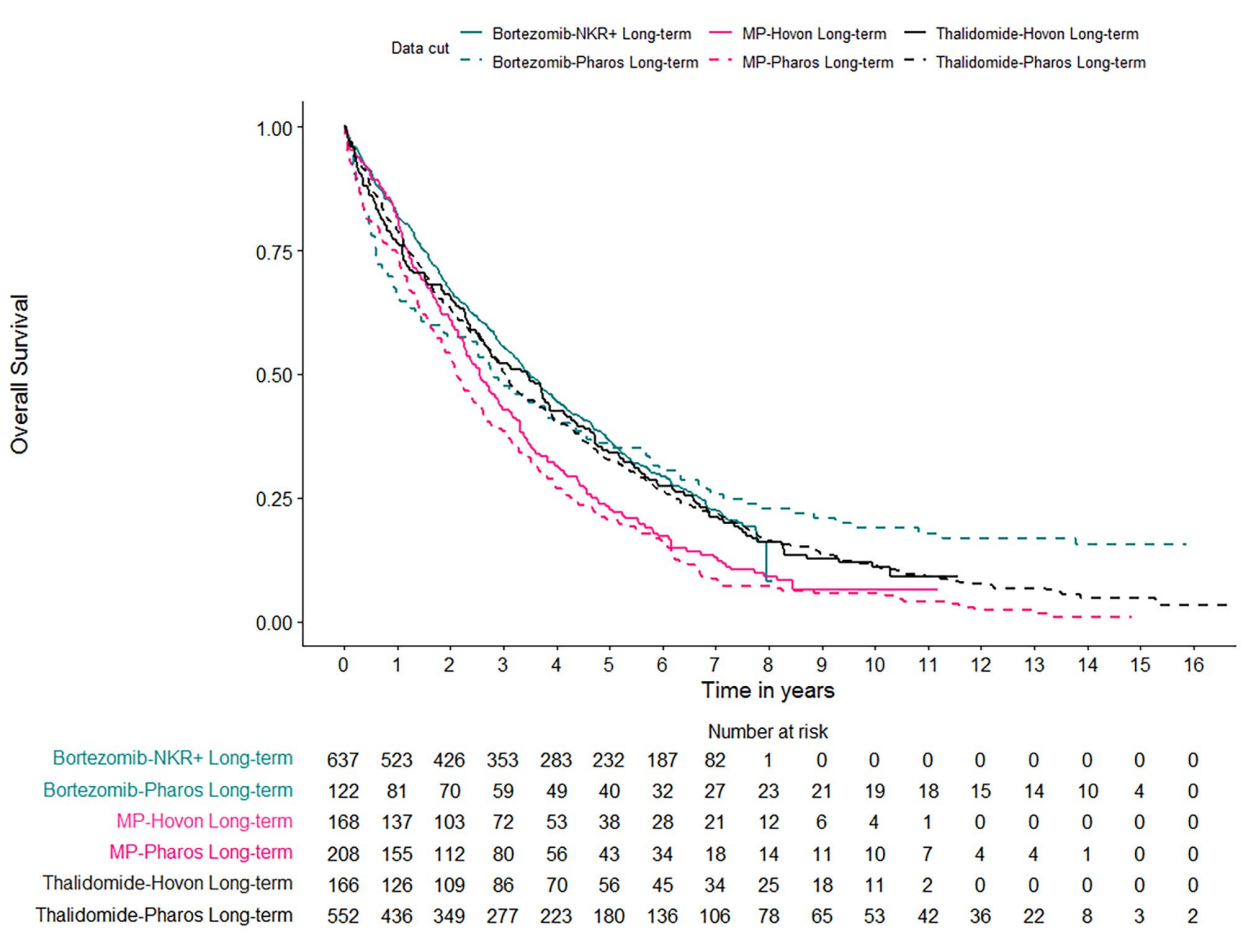
Long term overall survival of patients treated with bortezomib-based regimens for the PHAROS (registry) data and the NKR+ (registry) data, patients treated with MP-based regimens for the HOVON (RCT) data and the PHAROS (registry) data, and patients treated with thalidomide-based regimens for the HOVON (RCT) data and the PHAROS (registry) data

Overall, 1853 patients were included, who received a variety of treatment regimens in a regular clinical care setting (PHAROS & NKR+) or in an RCT (HOVON) (Table S1). For all patient cohorts the percentage censored was initially high but quickly decreased over time with longer follow-up (Table S1). Kaplan-Meier estimates and the number at risk for the respective time points were plotted grouped according to the treatment received (i.e., MP, thalidomide, bortezomib-based), the data source (i.e., HOVON, PHAROS, NKR+) and the maximum follow-up (i.e., 3, 6, 8, 10, and 13 years) (Fig. 1, S2-S7).

### Lifetime RMST

**Fig. 2:**
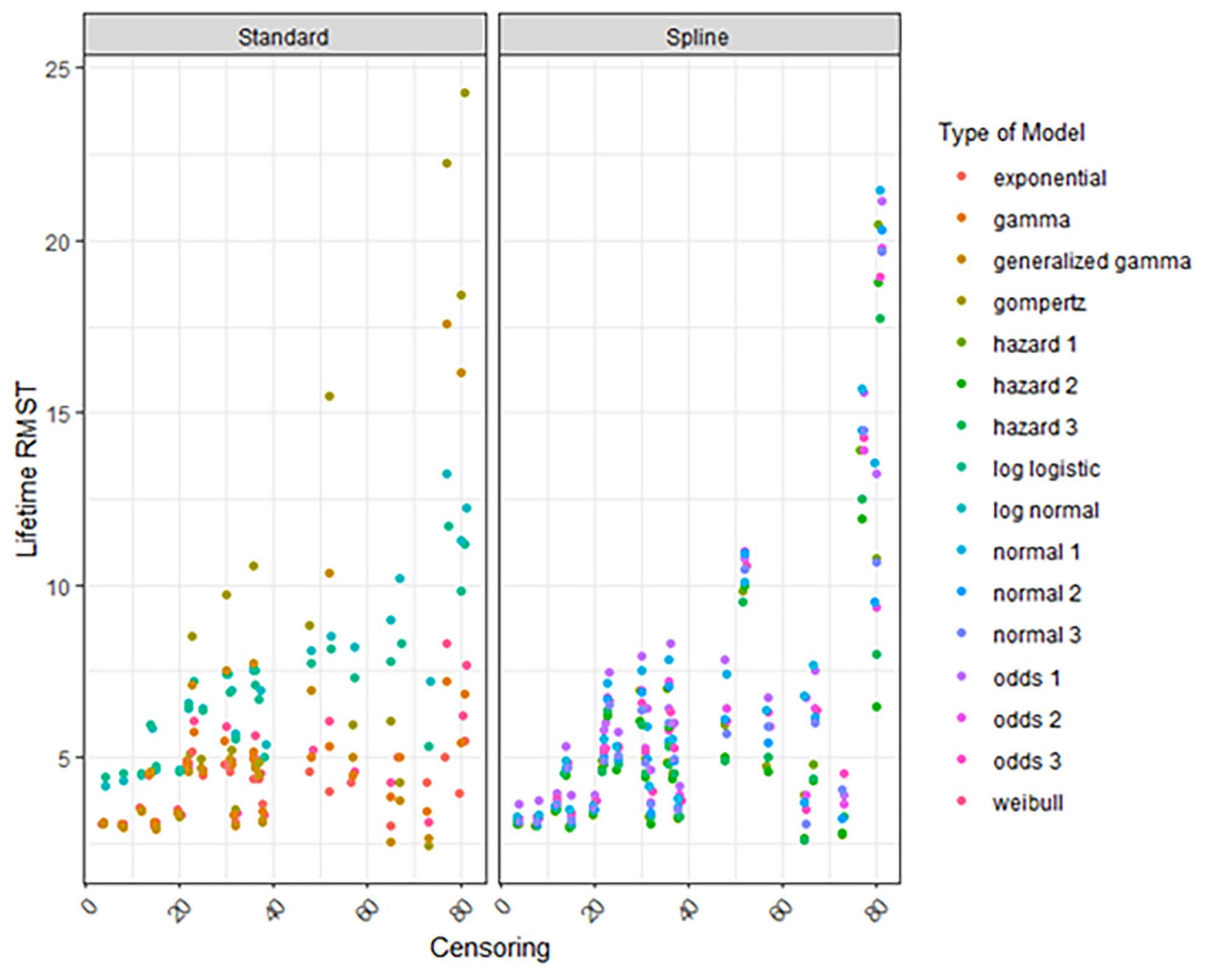
Lifetime RMST according to the percentage censored and the type of model

The extrapolated lifetime RMST varied considerably according to the data source and the types of models fitted (Figure S8). Overall, the variation in the extrapolated lifetime RMST was high for models estimated with limited follow-up. For example, for HOVON-Thal with a maximum follow-up of 3 years, the RMST varied from 5 years to 22.5 years. The variation for HOVON – MP, PHAROS – MP and, NKR+ - MPV was considerably smaller compared to all other arms (Figures S8, S9) varying from 2.5 years to less than 10 years. The survival estimates declined considerably as the percentage censored decreased (Fig. 2) but also as the absolute number of events increased for almost all models (Figure S10).

### Observed and estimated RMST from the RCT

In Table S2 we present a comparison between the observed long-term survival (i.e., 11 years) and estimated RMST for four different data cuts using data from the HOVON RCT. RMST estimates were restricted to the maximum follow-up. The mean survival estimates were considerably smaller compared to the 35-year time horizon, but the uncertainty was also large when follow-up was short. The standard parametric models were often selected based on AIC, BIC, and visual fit whereas no clear preference for either standard parametric models or spline models could be seen for the model with the lowest RMST error. Curves often overlapped and the differences between curves were often negligible making selections based on model fit difficult. We also observed that the RMST error based on the selection using BIC was almost always lower than the model selected based on AIC and visual fit. However, this was usually the exponential distribution which tended to under- or overestimate the hazard in the earlier months and vice versa in later months.

The RMST error was higher for the short-term follow-up (< 3 years) for which the censoring percentages were also higher (HOVON - MP: 73%, HOVON - Thal: 77%) relative to the number of events (HOVON - MP:29, HOVON - Thal: 25) (Table S1). However, as the length of follow-up increased, the error reduced with the absolute largest difference in RMST from < 3 years of follow-up to < 6 years of follow-up which coincided with a large reduction in censoring (HOVON - MP: 73–38%, HOVON - Thal: 77–48%). Confidence intervals of the models selected almost always overlapped.

### Observed and estimated RMST from registries

For the registries, the maximum follow-up was slightly longer and therefore the RMST was estimated for 14 years (Table S3). Here the model selected with the best visual fit seemed to change less often when follow-up increased, and standard parametric models were almost always selected based on AIC, BIC, and best visual fit. For the NKR + data, the absolute RMST error was much smaller due to shorter length of the time horizon for which RMST was estimated (i.e., 8 years).

Overall, standard parametric models regularly had the smallest absolute RMST error (i.e., in 67%) but as censoring decreased, the lowest absolute RMST error was more often a spline model (i.e., PHAROS - MP, NKR+ - MPV). The error in the extrapolations based on the datasets with short follow-up (< 3 years) was large, irrespective of the sample size of the dataset used and the percentage censored. The error decreased as the follow-up increased and thus censoring decreased.

### RMST error

**Fig. 3:**
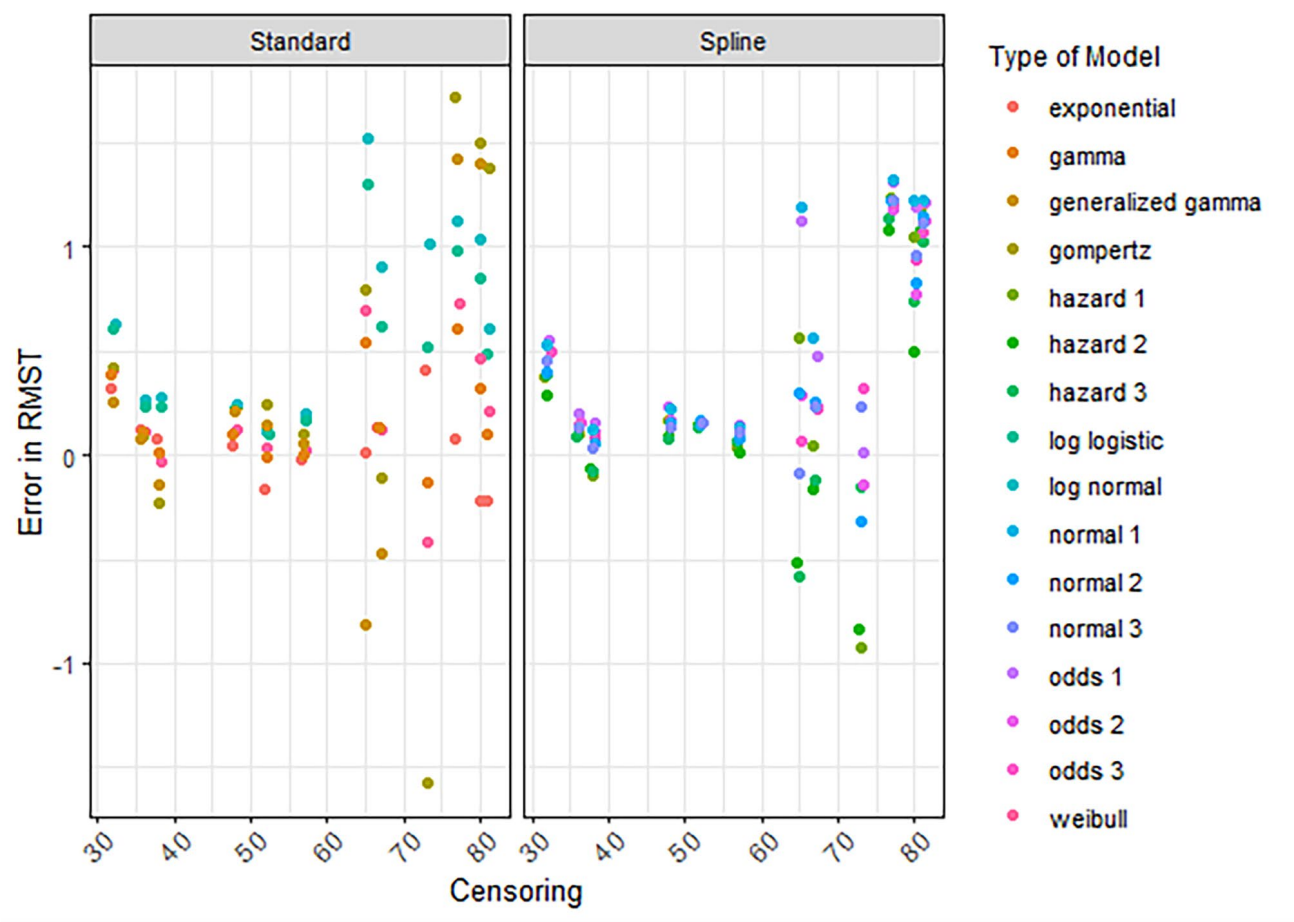
RMST error according to the percentage censored and the type of model. RMST is estimated for a time horizon of 8 years and a maximum follow-up of 3 and 6 years

The RMST error for all models decreased when follow-up increased (Figures S11-S14). The RMST errors for all treatments (regardless of the sample size, censoring, events, and the time horizon of the RMST) were low when 8 years of follow-up or more was available (S11-S14). Decreased censoring and more events coincided with smaller RMST errors (Fig. 3, S15-S17).

## RMST error conditional on survival

**Fig. 4:**
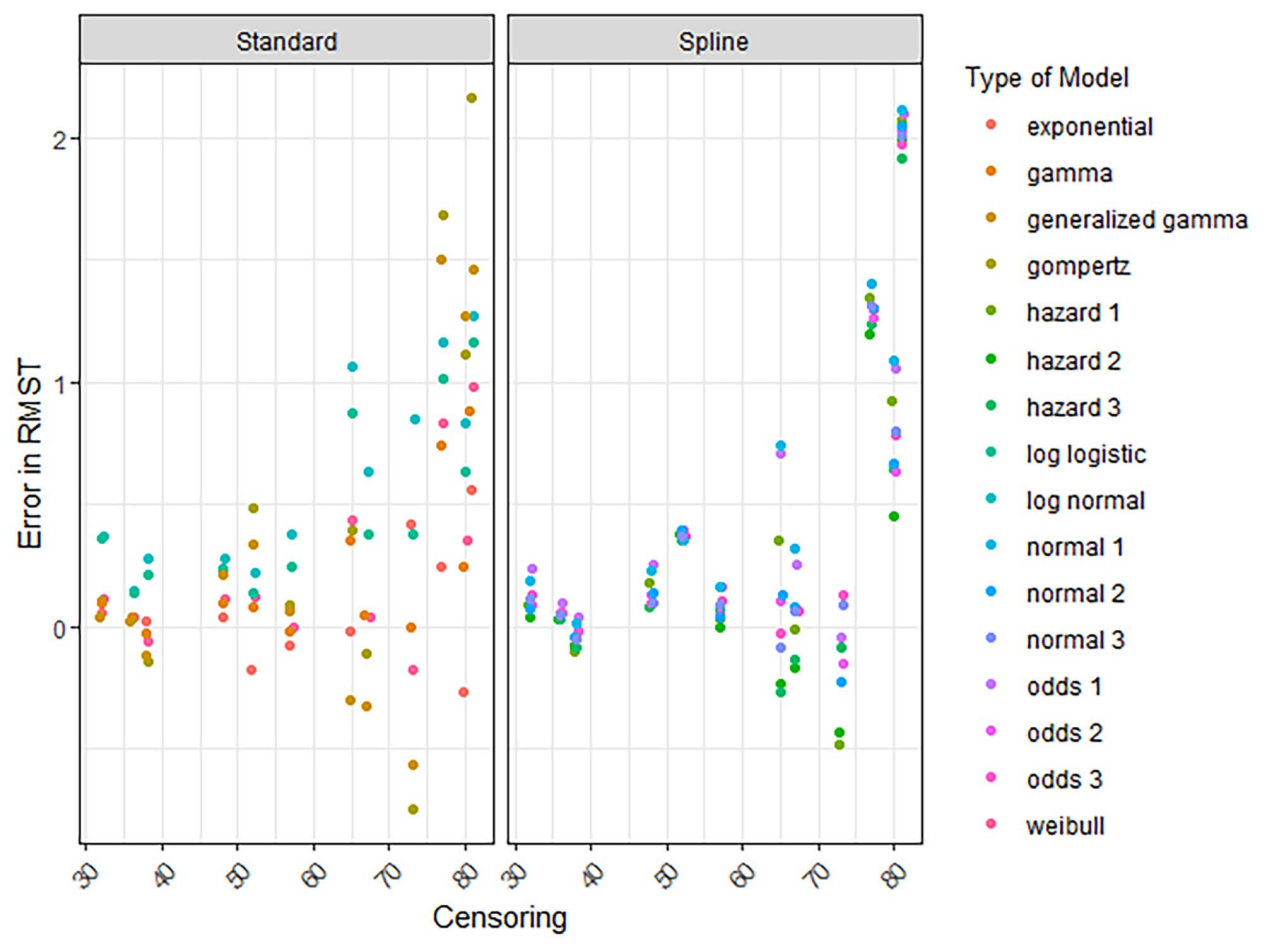
The RMST error conditional on surviving until extrapolation plotted according to the percentage censored and the type of model. RMST is estimated for a time horizon of 8 years and a maximum follow-up of 3 and 6 years

For the RMST error conditional on having survived until extrapolation, the decline was less pronounced as censoring reduced and the number of events increased (Fig. 4, S18). Moreover, the spread in error was much wider for standard parametric models compared to spline models (Fig. 4, S18, S19). In Fig. 4, the spread in the conditional RMST error between different models becomes smaller when censoring is less compared to the data cuts with higher percentages censored. However, even for the lowest percentages of censoring (e.g., 30–40%) there were some considerable deviations in the extrapolated RMST from the KM-estimate. This was also observed when the number of events was higher (e.g., > 100 events) (Figure S18).

## Discussion

In this study we analyze the accuracy of extrapolations for a non-solid tumor while varying the percentage censored using trial and registry data representative in sample size of those generally available to health economic researchers. We compared RMST estimated from extrapolated survival with the long-term Kaplan-Meier estimate in patients with multiple myeloma for a variety of treatments, data sources and maximum follow-up times. When reimbursement dossiers are drafted, the length of follow-up of patients included in the pivotal trial is often limited. Here, insight into the consequences of the uncertainty of extrapolations and the different models fitted, is essential since they are used to inform (conditional) reimbursement decisions of policy makers. This is an even bigger issue for clinical trials of novel immunotherapies such as daratumumab, where the percentage censored for overall survival is high [5, 26].

These results align with Everest et al., meaning that the RMST error increases when the percentage censored increases. For trials of hematologic diseases, the average percentage of censoring was 84% for initial publications and 54% for the final publication [4]. Although it can be seen in Fig. 3 and S15 that the RMST error has extensively declined for a percentage censored of 54% or lower, there can still be considerable uncertainty in these extrapolations. This was more pronounced when the error in RMST was estimated conditional on having survived until extrapolation (Fig. 4). Decision makers should critically review whether decisions on reimbursement can be made when the extrapolated survival is based on high percentages censored. In the economic evaluations that support these decisions, those models should be fitted which are relevant considering the disease at hand and based on clinical expertise. Here, the sensitivity analyses adopted by health economic researchers demonstrate the potential impact on cost-effectiveness of uncertainty for instance coinciding with high percentages censored but also when percentage censored is low.

In this study, we found no conclusive evidence that standard parametric models are better than spline models or vice versa. The highest absolute RMST error was regularly seen with a standard parametric model. This suggests that uncertainty analyses for health economic evaluations including all standard parametric models, could adequately address the extent (i.e., upper, and lower limits) of the uncertainty in the incremental cost effectiveness ratio. The relationship between the percentage censored and RMST error further stipulates the need to identify those methods that lead to the lowest RMST error, even when the percentage censored is high. Further research should assess whether spline models perform better or worse, with large percentages censored and a small absolute number of events.

### Limitations

This study focused on the RMST error as an outcome measure, which enables a comparison between the extrapolated and observed survival. There are however some drawbacks of this outcome measure. First, underestimation and overestimation over time can compensate and ultimately result in a relatively small RMST error. This aligns with results from a prior study in which large cohorts of registry data were used to extrapolate 10-year survival [9]. Gray et al. observed that the exponential distribution both under- and overestimated the hazard, resulting in a low RMST [9]. Second, for obtaining the RMST, a maximum time is required. While we could implement a life-time horizon for estimating RMST, we were bounded by the observation time for calculating the error in RMST which differed for the different data sources.

Another limitation of the outcome used is the fact that the Kaplan-Meier estimate itself is an estimate of the true survival function for a given cohort of patients. Although inherent to this kind of research, the error in RMST could be influenced by the fact that the number at risk decreases as time progresses. This is for instance reflected in the conditional survival estimated for HOVON-MP with > 10 years of follow-up where none of the few patients in the sample pass away between 10 and 11 years of follow-up.

Overall, the cohort size in our study was relatively small (i.e., smallest cohort of Gray et al. being N = 5407 [9]) which increases the uncertainty in extrapolated survival. This can also (partially) explain, why our findings differ from those by Gray et al. who found spline models to perform well even for short follow-up times. While larger cohorts are preferred and might be available for some treatments, our sample sizes are representative of clinical trials in hematology generally used as input for economic evaluations [5, 27, 28]. This makes our research applicable to current practices where health economic modelling is often performed using data from RCTs with a similar sample size. Another limitation was the heterogeneity in the PHAROS-bort cohort. The considerable uncertainty in the extrapolations for this cohort might be (partially) explained by the small sample size but perhaps also by the heterogeneity in the cohort. Due to the small sample size further stratification according to age was not feasible but would be recommended when such variation is present when performing an economic evaluation.

We employed commonly used parametric and spline models and did not consider more recent and complex models such as cure, parametric mixture, and landmark models [8, 15]. In Technical Support Document 21 from the National Institute for Health and Care Excellence Decision Support Unit, Rutherford et al. provide recommendations for their appropriate use and, although we did not include them in this analysis, they could be a relevant addition for instance when modelling survival for potentially curative treatments (e.g., CAR-T) [8]. Another topic for which an increasing amount of research is available concerns the inclusion of external data (e.g., registry data, national statistics). Including such external data to correct for excessively predicted survival in the extrapolations has been recommended when extrapolating survival from RCTs [7, 11]. Although this can sometimes reduce the overestimation of survival, this was beyond the scope of this study.

The generalizability of our findings to other areas of disease, particularly other hematological malignancies for which little evidence concerning the accuracy of extrapolations is available, will strongly depend on the similarities between the populations studied. The six datasets used in this study differ in the types of patients included, treatments administered, and hence in their hazard function. Similarly, the generalizability of these findings to other hematological malignancies will strongly depend on these features.

## Conclusions

In this study, we compare extrapolated survival of multiple myeloma patients to prolonged empirical survival for a wide variety of DCOs using data from an RCT and registries. Uncertainty in extrapolations can have a large impact on use of healthcare services when the error in long-term survival is large and when it leads to incorrect conclusions for decision makers.

We found that the RMST error can become quite small for both standard parametric and spline models but also that RMST error increases for all models as censoring increases. The error in RMST for the extrapolated period only also reduced as the percentage censored decreased and the number of events decreased. However, this reduction was much less pronounced.

Health economic researchers should consider a variety of models in their (uncertainty) analyses when extrapolating survival in economic evaluations. Here, although the RMST error is high when the percentage censored is high, careful consideration of uncertainty analyses also seems warranted when longer follow-up is available.

## Electronic supplementary material

Below is the link to the electronic supplementary material.

Supplementary Material 1

## Data Availability

The data that support the findings of this study are available in the Dutch Cancer Registry (IKNL), and the Dutch Haemato-oncology Foundation for Adults in the Netherlands (HOVON). Data are available upon reasonable request through the corresponding author (LB) under condition that permission for access is granted by the Dutch National Cancer Registry (IKNL), and the Dutch Haemato-oncology Foundation for Adults in the Netherlands (HOVON).

## List of Abbreviations

AIC: Akaike Information Criterion
AUC: Area under the curve
BIC: Bayesian Information Criterion
DCO: Data cut off
IPD: Individual patient data
HOVON: Dutch Haemato-Oncology Foundation for Adults in the Netherlands
LCI: Lower Confidence Interval
MP: Melphalan + Prednisone
NKR: Dutch National Cancer Registry
PHAROS: Dutch Population based HAematological Registry for Observational Studies
RCT: Randomized Controlled Trial
RMST: Restricted Mean Survival Time
UCI: Upper Confidence Interval
