# Supplementary material for: Extrapolating empirical long-term survival data: the impact of updated follow-up data and parametric extrapolation methods on survival estimates in multiple myeloma
Source: BMC Med Res Methodol. 2023 May 29;23:132. doi: 10.1186/s12874-023-01952-2 (PMC10226243; doi:10.1186/s12874-023-01952-2)

**Supplementary Information: Extrapolating empirical long-term survival data: the impact of updated follow-up data and parametric extrapolation methods on survival estimates in multiple myeloma**

LJ Bakker^1,2^, FW Thielen^1,2^, WK Redekop^1,2^, CA Uyl-de Groot^1,2^, HM Blommestein^1,2^

^1^ Erasmus School of Health Policy and Management, Erasmus University, Rotterdam, the Netherlands

^2^ Erasmus Centre for Health Economics Rotterdam, Erasmus University, Rotterdam, the Netherlands

**Corresponding author**: Lytske Bakker, MSc, ESHPM, Erasmus University Rotterdam, P.O. Box 1738, 3000 DR Rotterdam, the Netherlands; bakker@eshpm.eur.nl; Tel: +31 10 408 8867

Contents

[Figure S1. Figure demonstrating follow-up and inclusion for the different datasets and treatments. 4](#_Toc133174294)

[Table S1. Percentage right censored and the absolute number of events for the different data sources, treatments, and maximum follow-up. 5](#_Toc133174295)

[Figure S2. NKR+ Datasets- Bortezomib 6](#_Toc133174296)

[Figure S3. Pharos Datasets- Bortezomib 7](#_Toc133174297)

[Figure S4. Hovon Datasets- MP 8](#_Toc133174298)

[Figure S5. Pharos Datasets- MP 9](#_Toc133174299)

[Figure S6. Hovon Datasets - Thalidomide 10](#_Toc133174300)

[Figure S7. Pharos Datasets - Thalidomide 11](#_Toc133174301)

[Figure S8. Lifetime RMST according to data source, treatment, and follow-up 12](#_Toc133174302)

[Figure S9. Lifetime RMST according to data source, treatment, follow-up, and model complexity 13](#_Toc133174303)

[Figure S10. Lifetime RMST according to the absolute number of events and the type of model. 14](#_Toc133174304)

[Table S2: Restricted mean survival time (RMST) for the different treatments and data cut offs used for extrapolations from the HOVON RCT. 15](#_Toc133174305)

[Table S3: Restricted mean survival time (RMST) for different treatments, and data cut offs used for extrapolations from the PHAROS and NKR+ registries. 16](#_Toc133174306)

[Figure S11. Absolute Error in RMST according to data source, treatment, and follow-up 17](#_Toc133174307)

[Figure S12. Error in RMST according to data source, treatment, and follow-up 18](#_Toc133174308)

[Figure S13. Absolute Error in RMST according to data source, treatment, follow-up, and model complexity 19](#_Toc133174309)

[Figure S14. Error in RMST according to data source, treatment, follow-up, and model complexity 20](#_Toc133174310)

[Figure S15: Absolute RMST error according to the percentage censored and the type of model. The time horizon for RMST in this figure was 8 years for all datasets. 21](#_Toc133174311)

[Figure S16. Absolute Error in RMST according to the absolute number of events and the type of model restricted to 8 years of follow-up. 22](#_Toc133174312)

[Figure S17. Error in RMST according to the absolute number of events and the type of model restricted to 8 years of follow-up. 23](#_Toc133174313)

[Figure S18. Error in RMST conditional on survival until extrapolation according to the absolute number of events and the type of model. RMST is estimated for a time horizon of 8 years and a maximum follow-up of 3 and 6 years. 24](#_Toc133174314)

[Figure S19. Error in RMST conditional on survival until extrapolation according to data source, treatment, follow-up, and model. Here the spread for the Pharos data is much larger given that the time horizon is larger (14 years Pharos vs 11 years HOVON vs 8 years NKR+). 25](#_Toc133174315)

**Figure S1. Figure demonstrating follow-up and inclusion for the different datasets and treatments.**

**Table S1. Percentage right censored and the absolute number of events for the different data sources, treatments, and maximum follow-up.**

|  | HOVON | | PHAROS | | | NKR+ |
| --- | --- | --- | --- | --- | --- | --- |
| Treatment | MP | Thalidomide | MP | Thalidomide | Bortezomib | MPV |
| <3 years follow-up | | | | | | |
| N | 109 | 109 | 141 | 173 | 52 | 637 |
| Censored | 73% | 77% | 65% | 80% | 81% | 67% |
| Events | 29 | 25 | 50 | 35 | 10 | 210 |
| <6 years follow-up | | | | | | |
| N | 168 | 166 | 208 | 552 | 122 | 637 |
| Censored | 38% | 48% | 32% | 57% | 52% | 36% |
| Events | 104 | 87 | 142 | 240 | 59 | 405 |
| <8 years follow-up | | | | | | |
| N | 168 | 166 | 208 | 552 | 122 | 637 |
| Censored | 20% | 31% | 15% | 37% | 36% | 22% |
| Events | 134 | 114 | 176 | 350 | 78 | 494 |
| <10 years follow-up | | | | | | |
| N | 168 | 166 | 208 | 552 | 122 | - |
| Censored | 12% | 22% | 8% | 25% | 30% | - |
| Events | 148 | 130 | 191 | 415 | 86 | - |
| <13 years follow-up | | | | | | |
| N | - | - | 208 | 552 | 122 | - |
| Censored | - | - | 4% | 14% | 23% | - |
| Events | - | - | 199 | 472 | 94 | - |

**Figure S2. NKR+ Datasets- Bortezomib**

a. <3 years follow-up b. <6 years follow-up


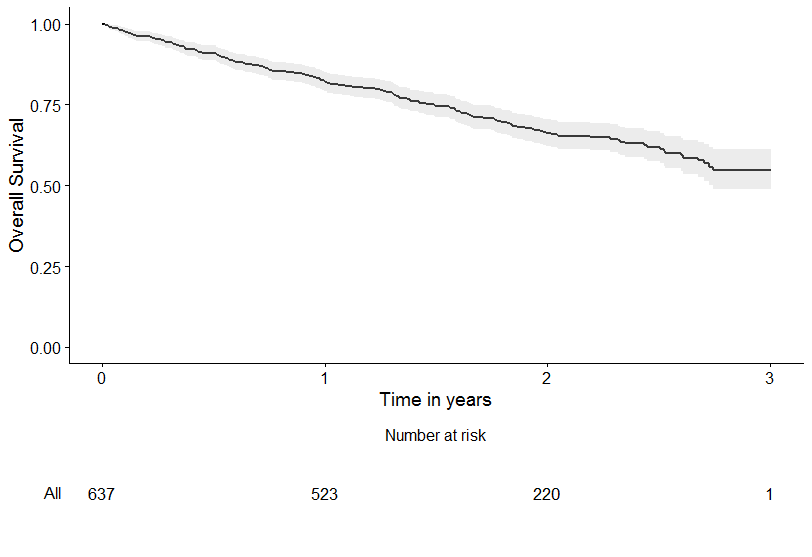

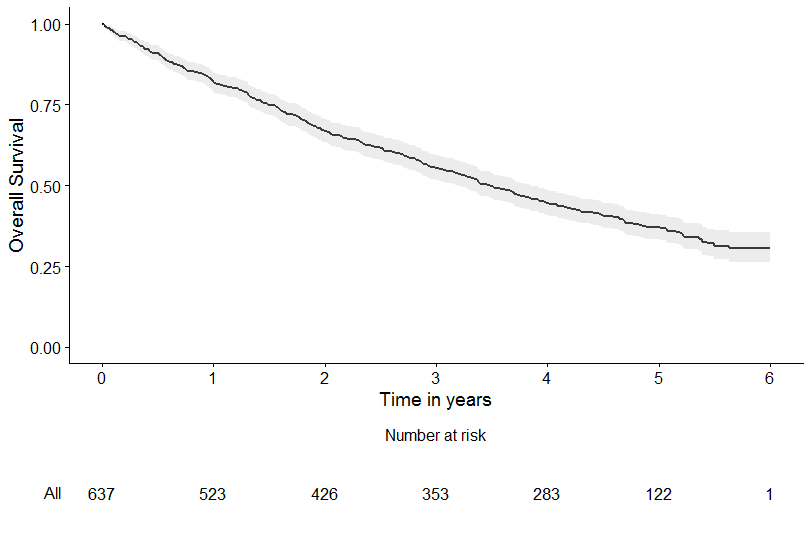


c. <8 years follow-up d. Long-term follow-up


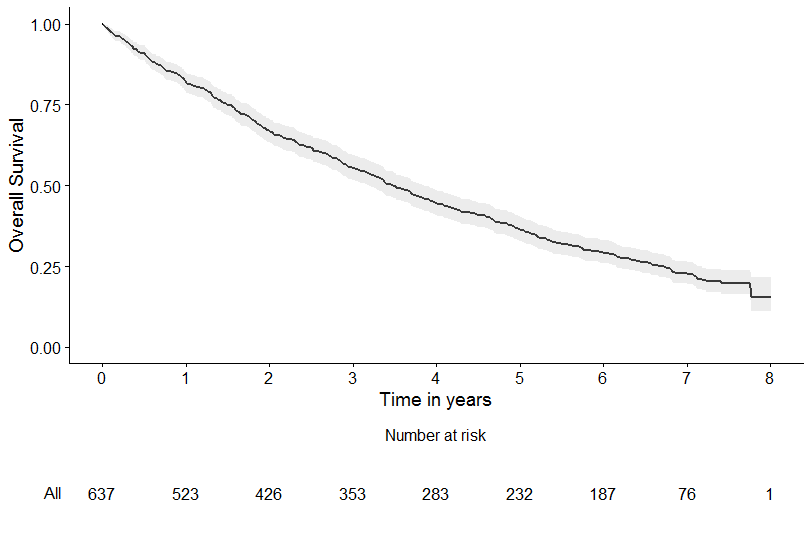

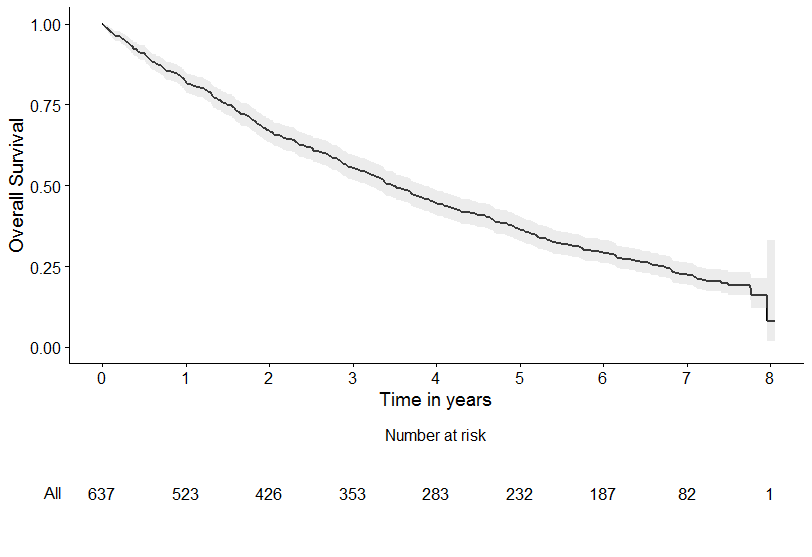


**Figure S3. Pharos Datasets- Bortezomib**

a. <3 years follow-up b. <6 years follow-up


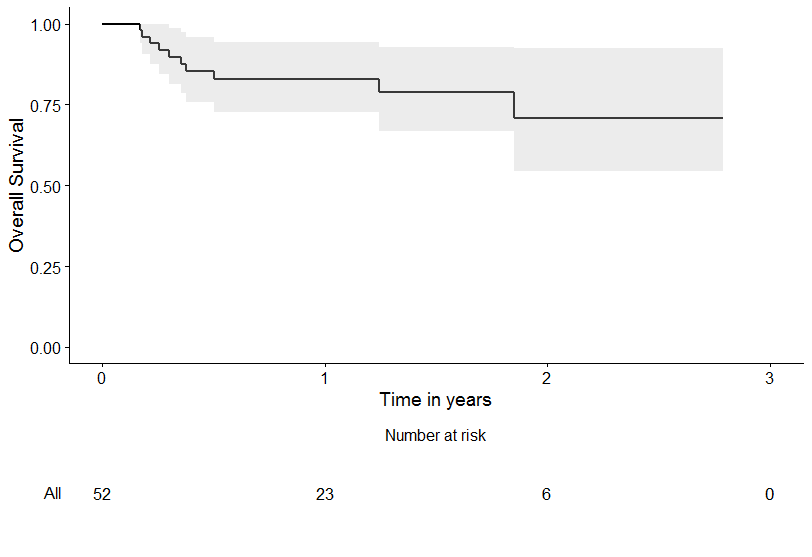

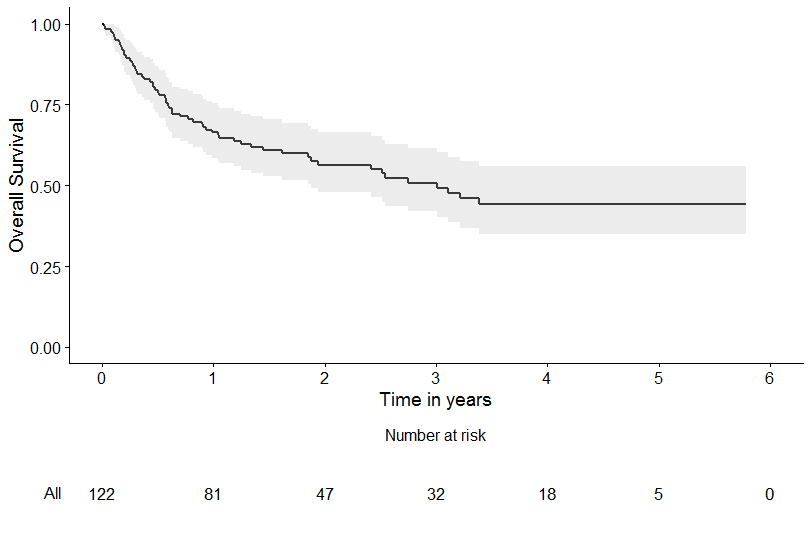


c. <8 years follow-up d. <10 years follow-up


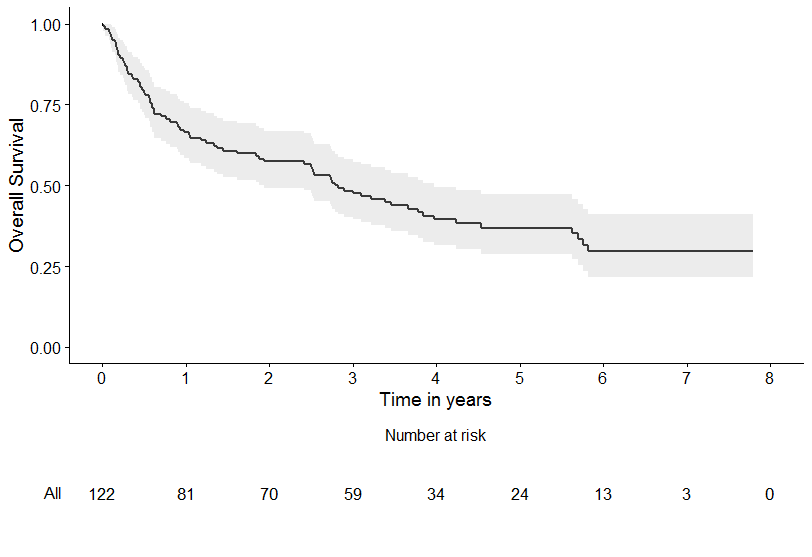

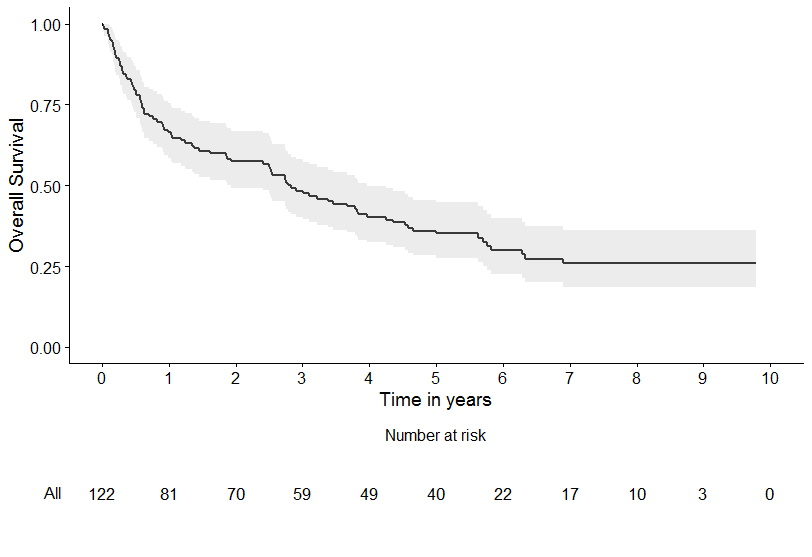


e. <13 years follow-up f. Long-term follow-up


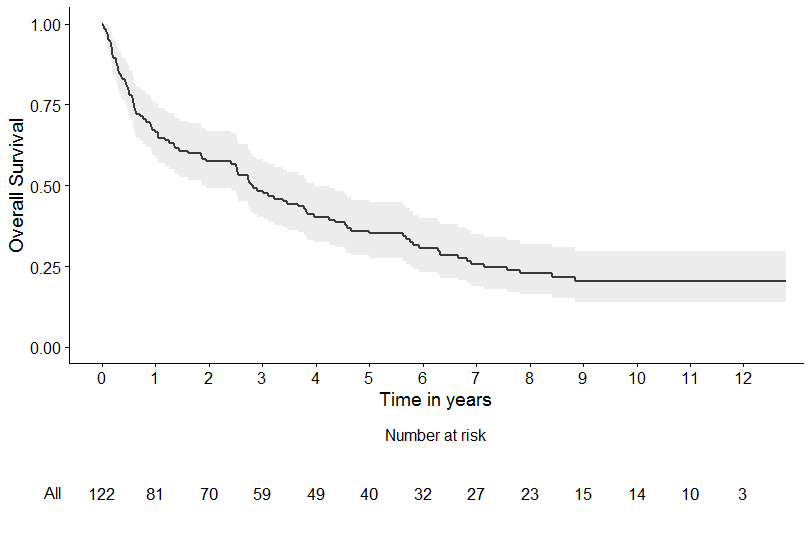

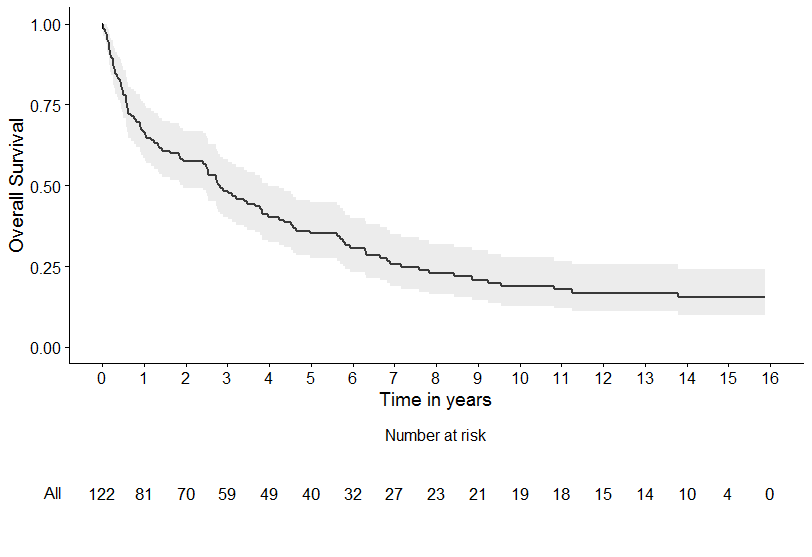


**Figure S4. Hovon Datasets- MP**

a. <3 years follow-up b. <6 years follow-up


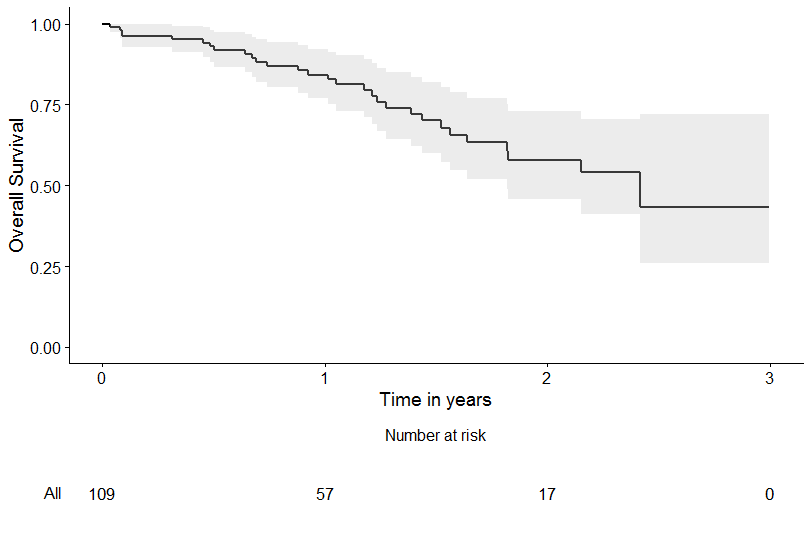

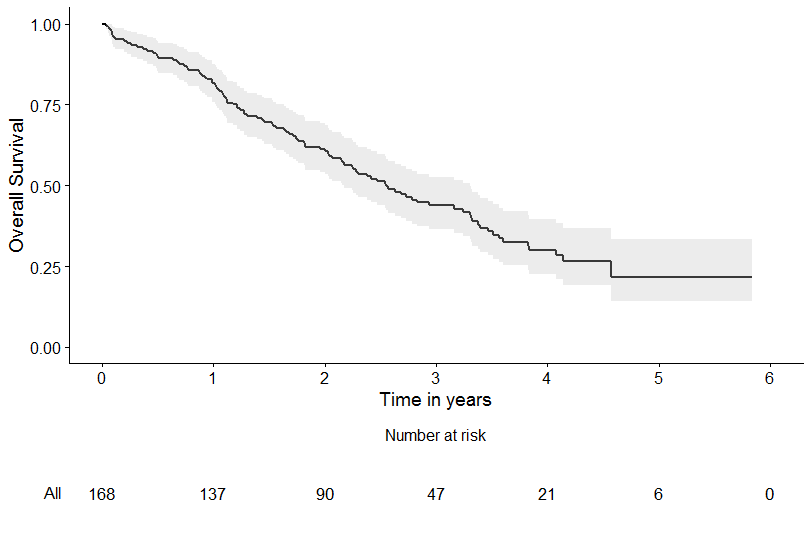


c. <8 years follow-up d. <10 years follow-up


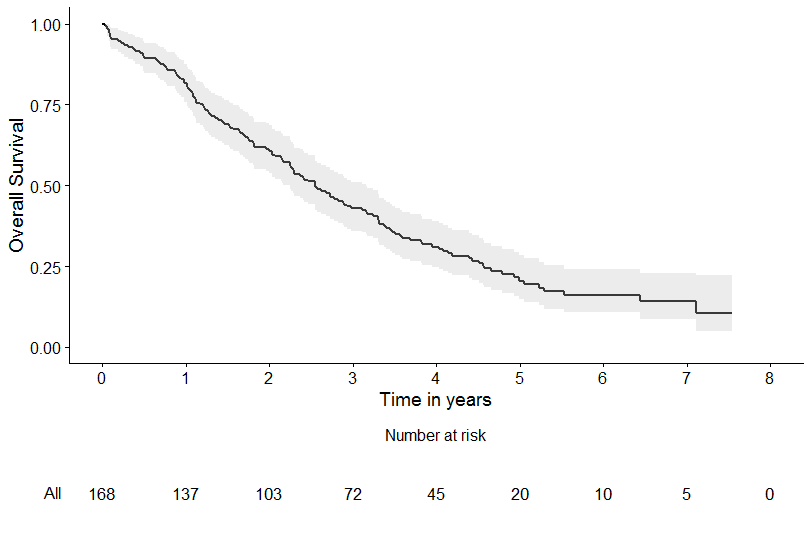

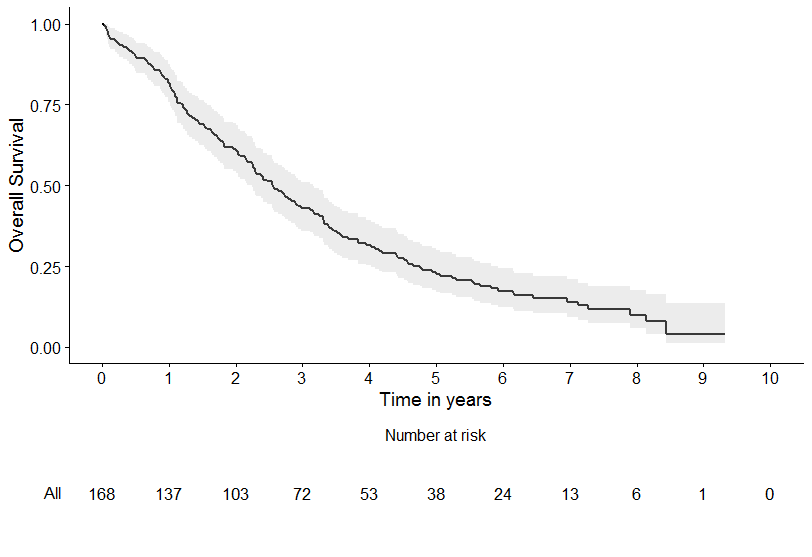


e. Long-term follow-up


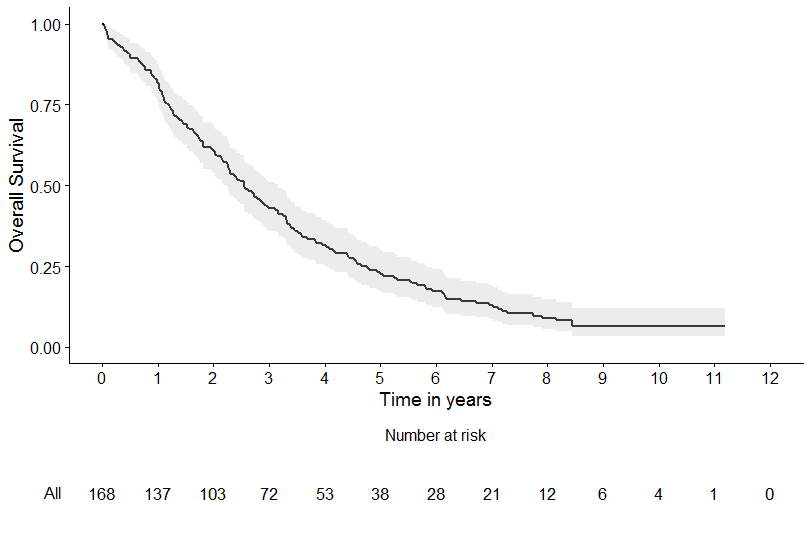


**Figure S5. Pharos Datasets- MP**

a. <3 years follow-up b. <6 years follow-up


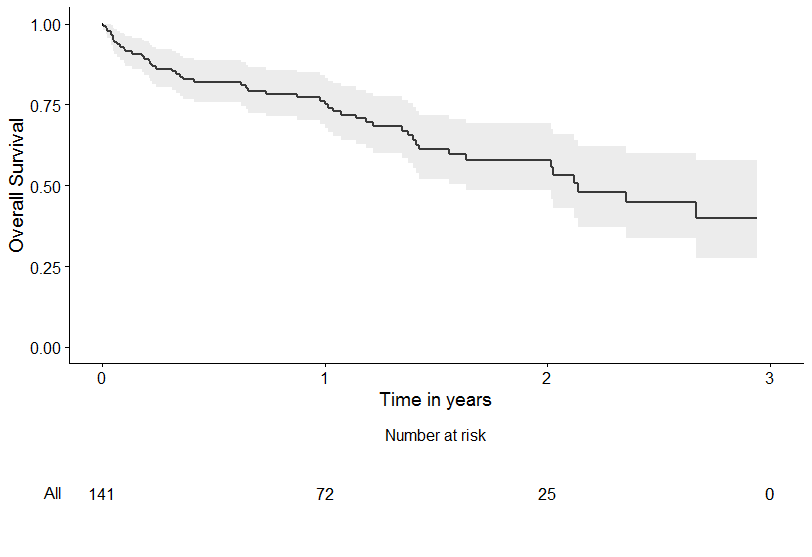

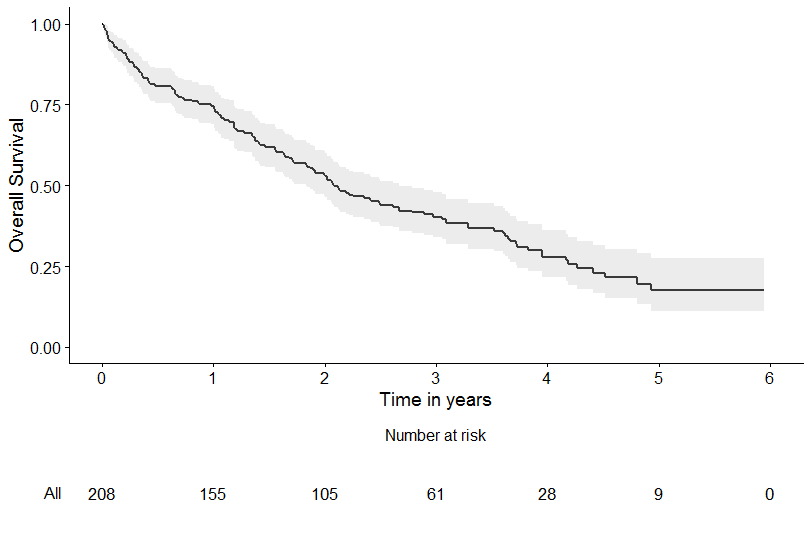


c. <8 years follow-up d. <10 years follow-up


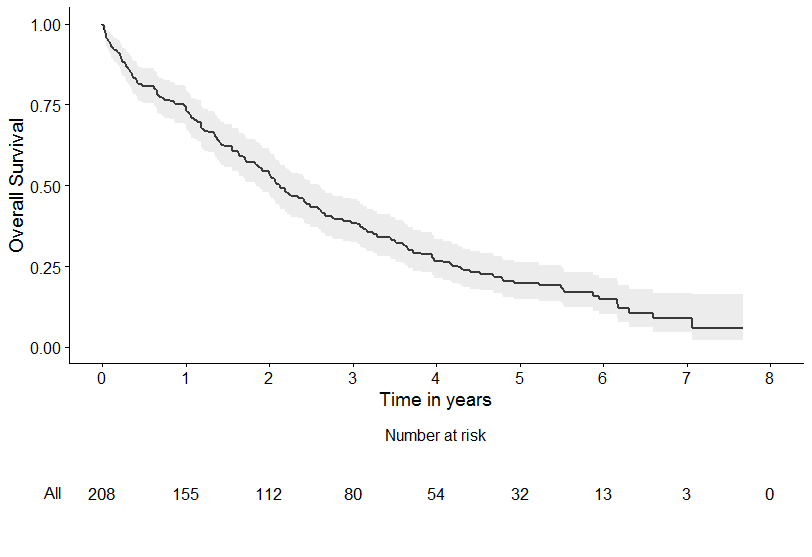

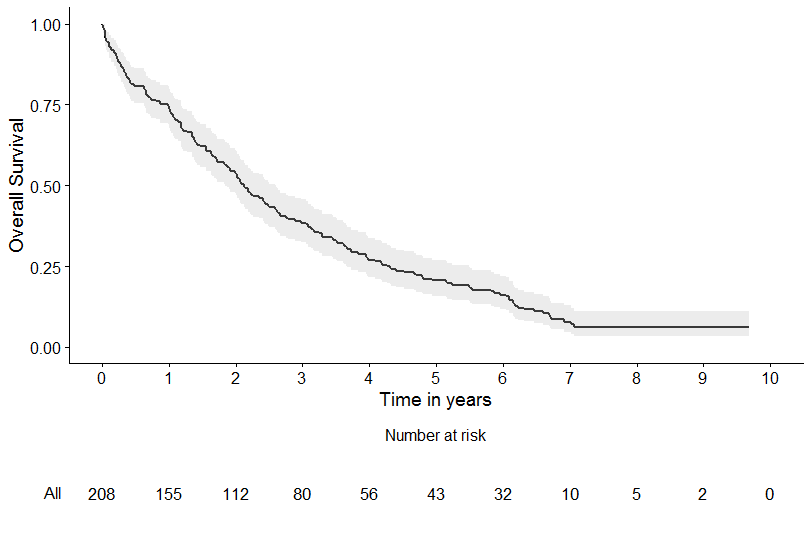


e. <13 years follow-up f. Long-term follow-up


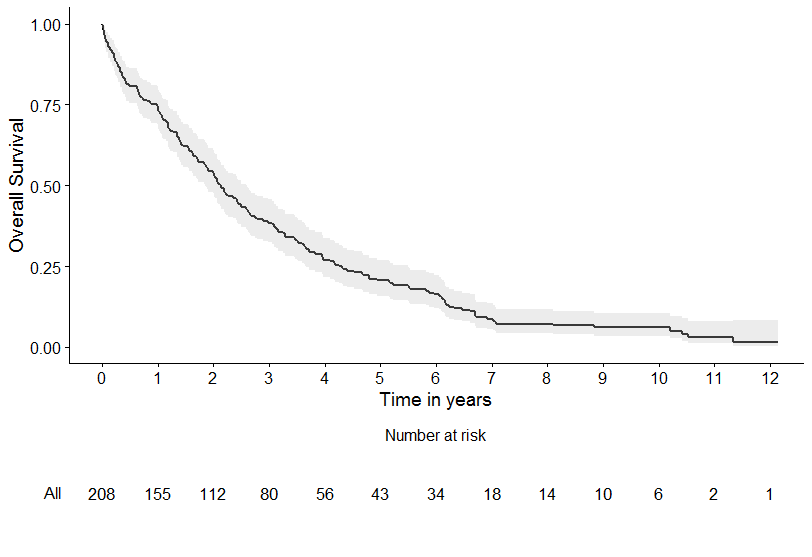

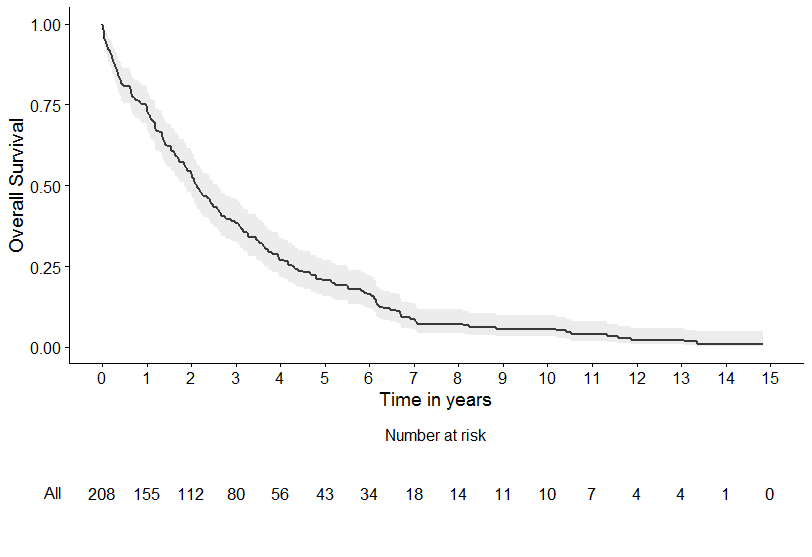


**Figure S6. Hovon Datasets - Thalidomide**

a. <3 years follow-up b. <6 years follow-up


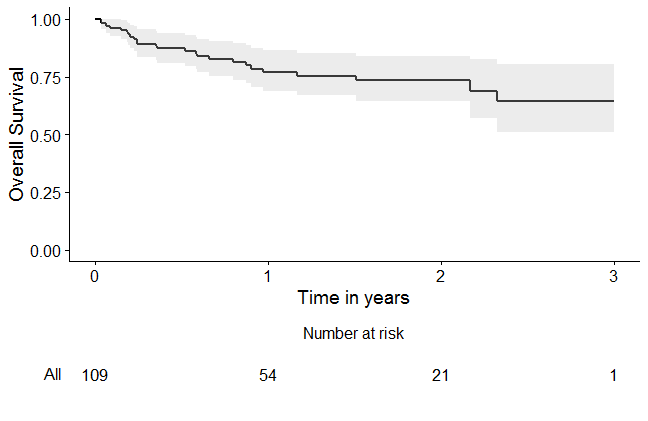

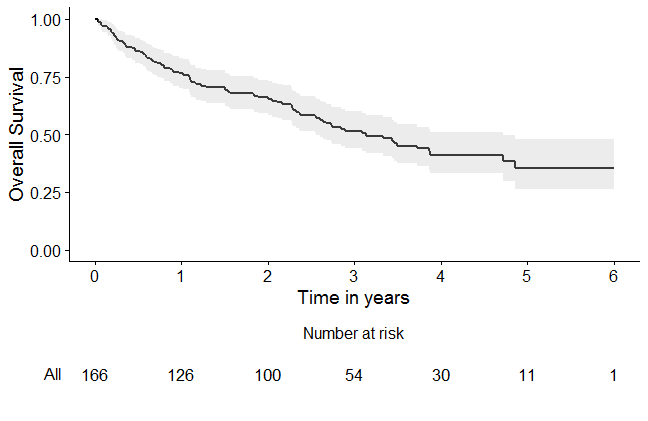


c. <8 years follow-up d. <10 years follow-up


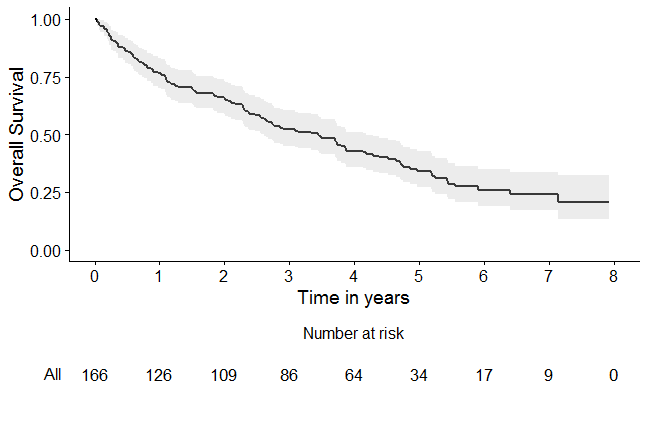

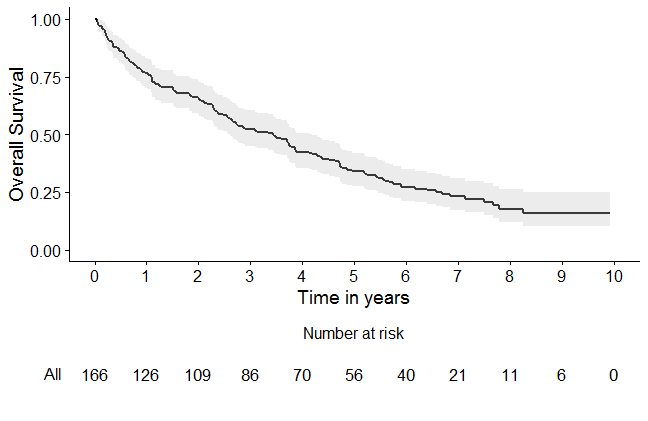


e. Long-term follow-up


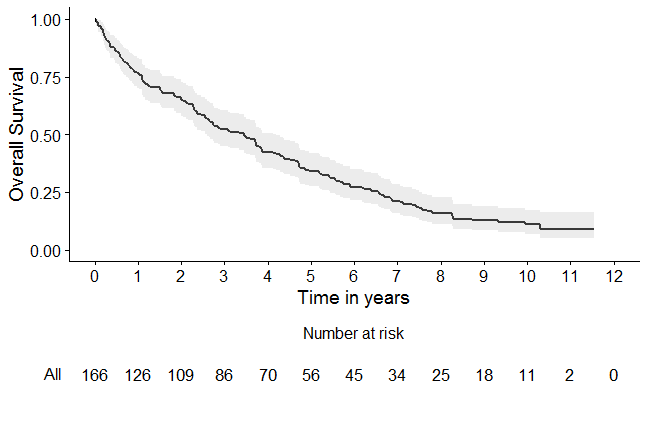


**Figure S7. Pharos Datasets - Thalidomide**

a. <3 years follow-up b. <6 years follow-up


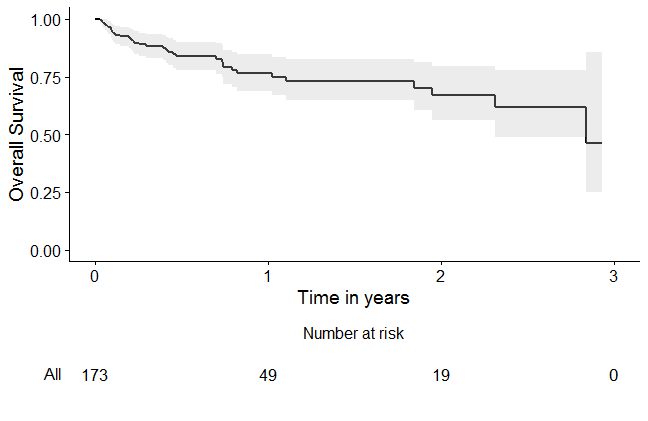

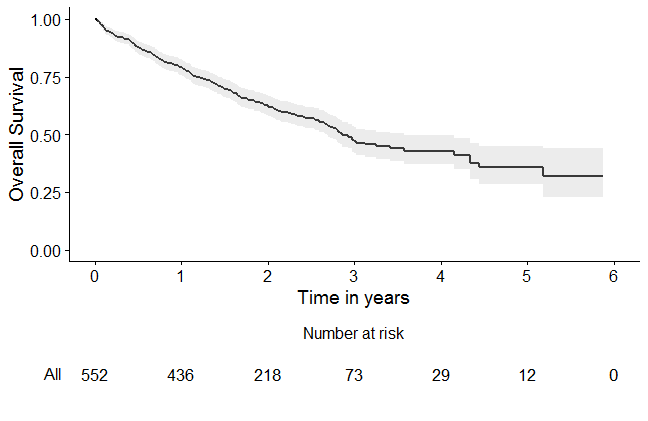


c. <8 years follow-up d. <10 years follow-up


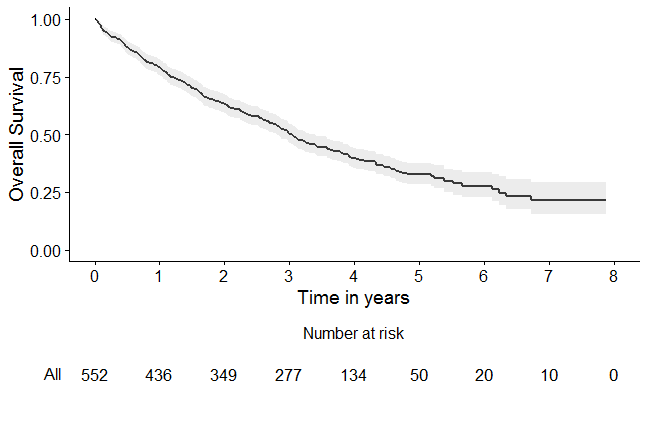

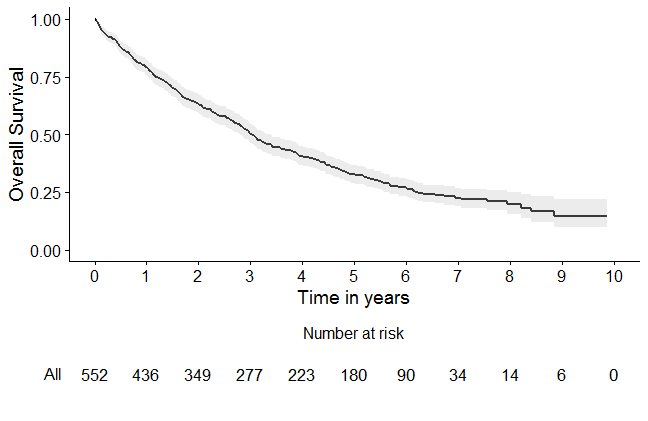


e. <13 years follow-up f. Long-term follow-up

**
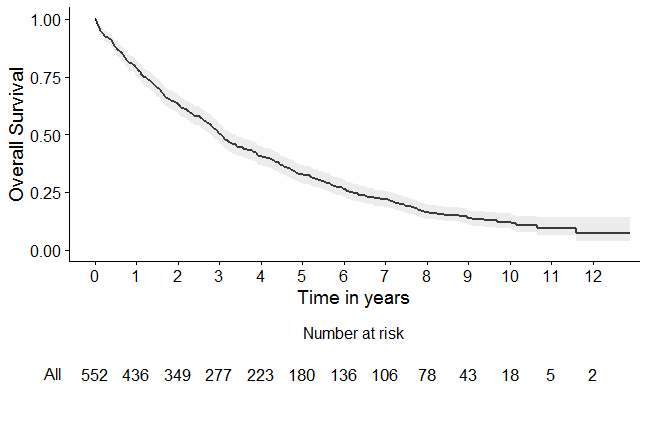

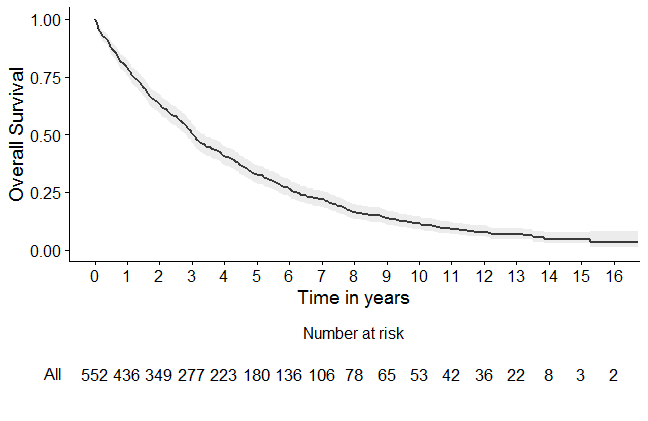
**

**Figure S8. Lifetime RMST according to data source, treatment, and follow-up**


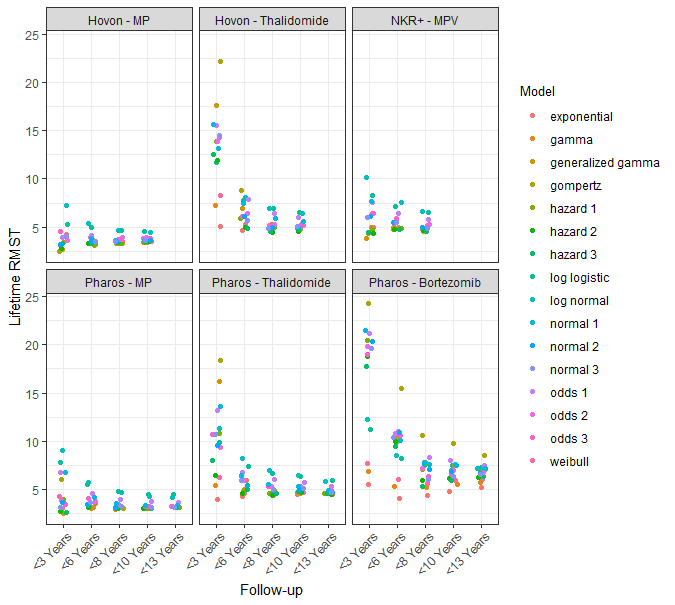


**Figure S9. Lifetime RMST according to data source, treatment, follow-up, and model complexity**


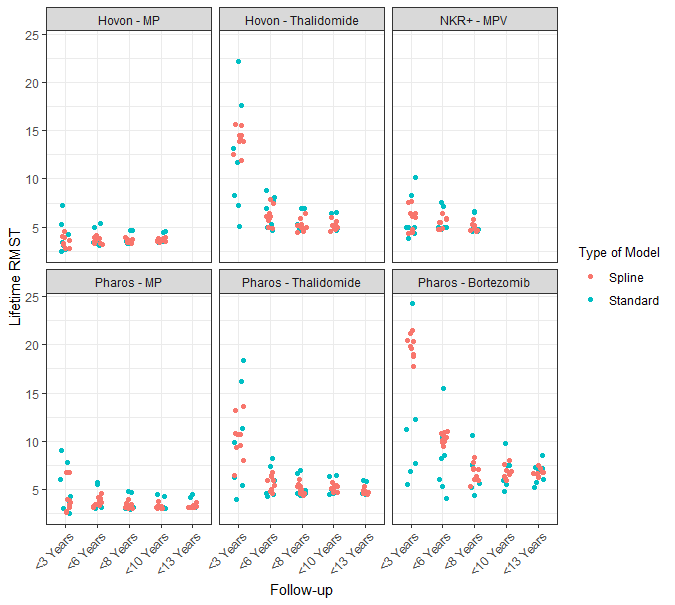


**Figure S10. Lifetime RMST according to the absolute number of events and the type of model.**


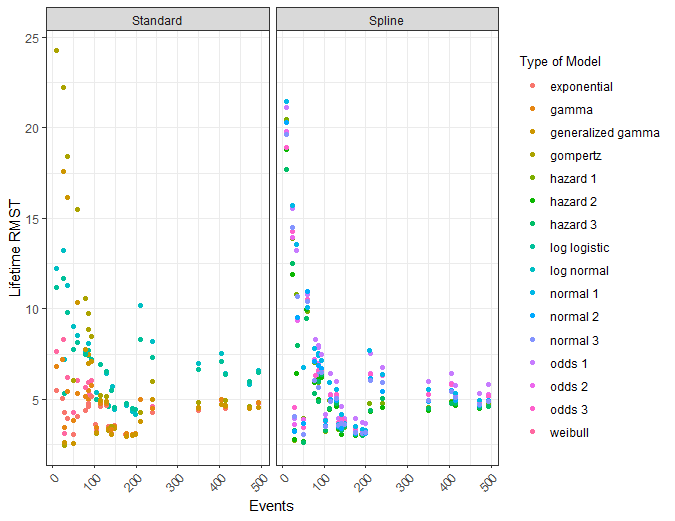


**Table S2: Restricted mean survival time (RMST) for the different treatments and data cut offs used for extrapolations from the HOVON RCT.**

|  |  | HOVON - MP | | | HOVON - Thal | | |
| --- | --- | --- | --- | --- | --- | --- | --- |
|  | Follow-up | Model | RMST (LCI; UCI) | Error | Model | RMST (LCI; UCI) | Error |
| Lowest AIC | <3Y | Gompertz | 2.45(1.91;4.60) | -0.95 | LogNormal | 6.21(4.48;7.39) | 2.10 |
| Lowest BIC | <3Y | Exponential | 4.27(2.97;5.94) | 0.87 | LogNormal | 6.21(4.48;7.39) | 2.10 |
| Best visual fit | <3Y | Weibull | 3.11(2.10;4.78) | -0.29 | LogNormal | 6.21(4.48;7.39) | 2.10 |
| Lowest error RMST | <3Y | Gamma | 3.42(2.32;5.21) | 0.02 | Exponential | 4.46(3.30;5.75) | 0.35 |
| Lowest AIC | <6Y | Gompertz | 3.11 (2.67;3.68) | -0.30 | Log Normal | 4.83 (4.02; 5.53) | 0.72 |
| Lowest BIC | <6Y | Exponential | 3.45 (2.94;4.01) | 0.05 | Exponential | 4.19 (3.56; 4.85) | 0.07 |
| Best visual fit | <6Y | Gompertz | 3.11 (2.67;3.68) | -0.30 | Weibull | 4.39 (3.58; 5.05) | 0.28 |
| Lowest error RMST | <6Y | Odds 2 | 3.42 (2.93;4.08) | 0.02 | Exponential | 4.19 (3.56; 4.85) | 0.07 |
| Lowest AIC | <8Y | Weibull | 3.29 (2.84;3.75) | -0.11 | Normal 2 | 4.21(3.66; 4.88) | 0.10 |
| Lowest BIC | <8Y | Exponential | 3.35 (2.88;3.85) | -0.05 | Exponential | 4.19 (3.61; 4.77) | 0.07 |
| Best visual fit | <8Y | Gamma | 3.31 (2.88;3.78) | -0.09 | Weibull | 4.24 (3.65; 4.83) | 0.12 |
| Lowest error RMST | <8Y | Normal 1 | 3.37(2.92;3.90) | -0.03 | Hazard 2 | 4.13 (3.55;4.77) | 0.01 |
| Lowest AIC | <10Y | Weibull | 3.38 (2.99;3.84) | -0.02 | Normal 2 | 4.22 (3.70; 4.80) | 0.11 |
| Lowest BIC | <10Y | Exponential | 3.38 (2.90;3.88) | -0.02 | Exponential | 4.23 (3.66; 4.79) | 0.12 |
| Best visual fit | <10Y | Hazard 2 | 3.39 (2.98;3.83) | -0.01 | Weibull | 4.23 (3.68; 4.75) | 0.11 |
| Lowest error RMST | <10Y | Normal 2* | 3.40 (2.98;3.89) | 0.00 | Hazard 2 | 4.21 (3.64;4.76) | 0.09 |
| RMST KM | 3.40 (2.96;3.84) | | | | 4.12 (3.59;4.64) | | |
| AIC= Akaike Information Criterion, BIC= Bayesian Information Criterion, abs.=absolute, HOVON= Dutch Haemato-oncology Foundation for Adults in the Netherlands, KM= Kaplan-Meier, LCI=Lower Confidence Interval, MP= melphalan+ prednisone, RMST=Restricted mean survival time, Thal.= Thalidomide based, UCI= Upper Confidence Interval, * Normal 3 equally small | | | | | | | |

**Table S3: Restricted mean survival time (RMST) for different treatments, and data cut offs used for extrapolations from the PHAROS and NKR+ registries.**

|  | | PHAROS - MP | | | PHAROS - Thal | | | PHAROS- Bort | | | NKR+ - MPV | | |
| --- | --- | --- | --- | --- | --- | --- | --- | --- | --- | --- | --- | --- | --- |
|  | **Follow-up** | **Model** | **RMST (95%CI)** | **Error** | **Model** | **RMST (95%CI)** | **Error** | **Model** | **RMST (95%CI)** | **Error** | **Model** | **RMST (95%CI)** | **Error** |
| Lowest AIC | <3Y | Odds 2 | 3.38(2.43;5.01) | 0.31 | LogNormal | 6.59(4.56;8.11) | 2.31 | Normal 2 | 9.31(5.94;12.16) | 4.54 | Exponential | 4.00(3.65;4.28) | 0.05 |
| Lowest BIC | <3Y | Exponential | 3.01(2.30;3.93) | -0.06 | LogNormal | 6.59(4.56;8.11) | 2.31 | Exponential | 5.06(2.83;7.83) | 0.30 | Exponential | 4.00(3.65;4.28) | 0.05 |
| Best visual fit | <3Y | Gamma | 3.66(2.47;5.03) | 0.59 | Gamma | 4.85(3.01;6.60) | 0.57 | Gamma | 5.81(2.25;8.71 | 1.05 | Gamma | 4.00(3.62;4.33) | 0.05 |
| Lowest error RMST | <3Y | Exponential | 3.01(2.30;3.93) | -0.06 | Exponential | 3.84(2.78;5.27) | -0.44 | Exponential | 5.06(2.83;7.83) | 0.30 | Hazard 1 | 3.94(3.52;4.36) | -0.01 |
| Lowest AIC | <6Y | Gamma | 3.24(2.71;3.79) | 0.17 | Exponential | 4.13(3.71;4.59) | -0.16 | Gompertz | 6.67(4.95;7.85) | 1.91 | Exponential | 3.99(3.76;4.22) | 0.04 |
| Lowest BIC | <6Y | Exponential | 3.10(2.64;3.63) | 0.03 | Exponential | 4.13(3.71;4.59) | -0.16 | Gompertz | 6.67(4.95;7.85) | 1.91 | Exponential | 3.99(3.76;4.22) | 0.04 |
| Best visual fit | <6Y | Weibull | 3.29(2.72;3.91) | 0.22 | Gompertz | 4.69(3.62;5.86) | 0.41 | GenGamma | 5.82(4.50;7.34) | 1.06 | Gompertz | 3.98(3.75;4.21) | 0.03 |
| Lowest error RMST | <6Y | Hazard 2 | 3.06 (2.62;3.80) | -0.01 | Gamma | 4.27(3.75;4.80) | -0.02 | Weibull | 4.86(3.61;6.10) | 0.10 | GenGamma | 3.97(3.74;4.22) | 0.02 |
| Lowest AIC | <8Y | GenGamma | 2.93(2.59;3.47) | -0.14 | Exponential | 4.21(3.87;4.60) | -0.08 | LogNormal | 5.02(4.04;6.00) | 0.26 | Gompertz | 3.96(3.72;4.17) | 0.01 |
| Lowest BIC | <8Y | Exponential | 3.01(2.57;3.46) | -0.06 | Exponential | 4.21(3.87;4.60) | -0.08 | LogNormal | 5.02(4.04;6.00) | 0.26 | Exponential | 3.91(3.70;4.12) | -0.04 |
| Best visual fit | <8Y | Weibull | 3.06(2.65;3.54) | -0.01 | Gompertz | 4.37(3.84;4.95) | 0.09 | Hazard 2 | 4.75(3.75;5.85) | -0.01 | Gompertz | 3.96(3.72;4.17) | 0.01 |
| Lowest error RMST | <8Y | Weibull | 3.06(2.65;3.54) | -0.01 | GenGamma | 4.29(3.85;4.84) | 0.00 | Hazard 2 | 4.75(3.75;5.85) | -0.01 | Hazard 1 | 3.95(3.75;4.15) | -0.00 |
| Lowest AIC | <10Y | GenGamma | 2.98(2.62;3.44) | -0.09 | Weibull | 4.35(3.98;4.75) | 0.07 | LogNormal | 4.97(4.10;6.00) | 0.21 | - | - | - |
| Lowest BIC | <10Y | Exponential | 3.01(2.60;3.44) | -0.06 | Exponential | 4.31(3.97;4.66) | 0.02 | LogNormal | 4.97(4.10;6.00) | 0.21 | - | - | - |
| Best visual fit | <10Y | Weibull | 3.03(2.62;3.44) | -0.04 | Gompertz | 4.41(4.00;4.84) | 0.13 | GenGamma | 4.98(4.04;6.04) | 0.22 | - | - | - |
| Lowest error RMST | <10Y | Odds 3 | 3.07(2.67;3.59) | 0.00 | Exponential | 4.31(3.97;4.66) | 0.02 | Gamma | 4.76(3.82;5.64) | -0.01 | - | - | - |
| Lowest AIC | <13Y | Gamma | 3.06(2.63;3.54) | -0.02 | Exponential | 4.28 (3.97;4.59) | -0.00 | Odds 2 | 4.84(4.04;5.76) | 0.08 | - | - | - |
| Lowest BIC | <13Y | Exponential | 3.06(2.68;3.48) | -0.01 | Exponential | 4.28 (3.97;4.59) | -0.00 | LogNormal | 4.87(3.94;5.79) | 0.10 | - | - | - |
| Best visual fit | <13Y | Weibull | 3.06(2.66;3.47) | -0.01 | Weibull | 4.29 (3.97;4.64) | 0.00 | GenGamma | 4.86(3.93;5.84) | 0.09 | - | - | - |
| Lowest error RMST | <13Y | Odds 3 | 3.08(2.68;3.59) | 0.01 | Gamma | 4.29(3.96;4.62) | 0.00 | Exponential | 4.83(4.11;5.59) | 0.06 | - | - | - |
| RMST KM* |  | 3.07(2.66;3.48) | | | 4.29(3.96;4.61) | | | 4.76(3.88;5.65) | | | 3.95 (3.74;4.16) | | |
| AIC= Akaike Information Criterion, BIC= Bayesian Information Criterion, Bort. = bortezomib, KM= Kaplan-Meier, LCI=Lower Confidence Interval, MP= melphalan+ prednisone, MPV= melphalan+ prednisone+ bortezomib, NKR+= Dutch National Cancer Registry, PHAROS= Population based HAematological Registry for Observational Studies, RMST=Restricted mean survival time, Thal. = Thalidomide based, UCI= Upper Confidence Interval * Time horizon PHAROS RMST: 14 years, Time horizon NKR+ RMST: 8 years. | | | | | | | | | | | | | |

**Figure S11. Absolute Error in RMST according to data source, treatment, and follow-up**


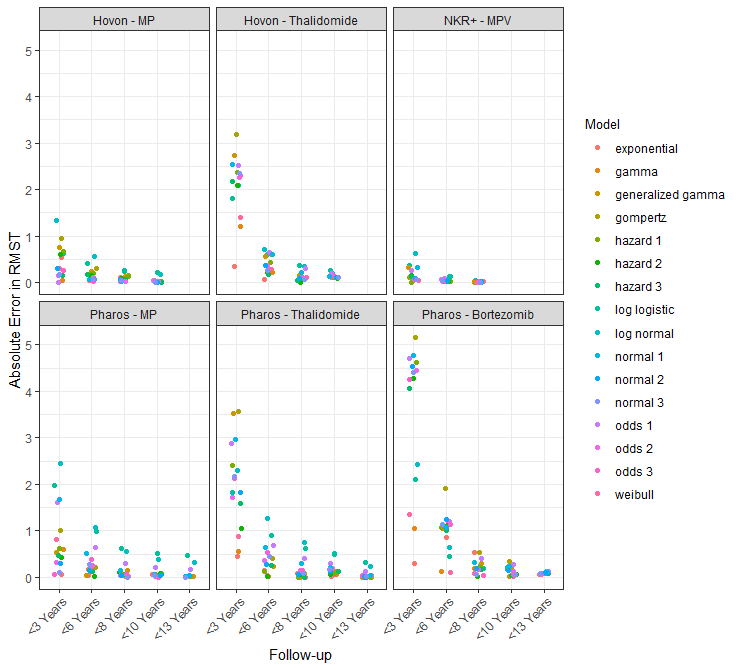


**Figure S12. Error in RMST according to data source, treatment, and follow-up**


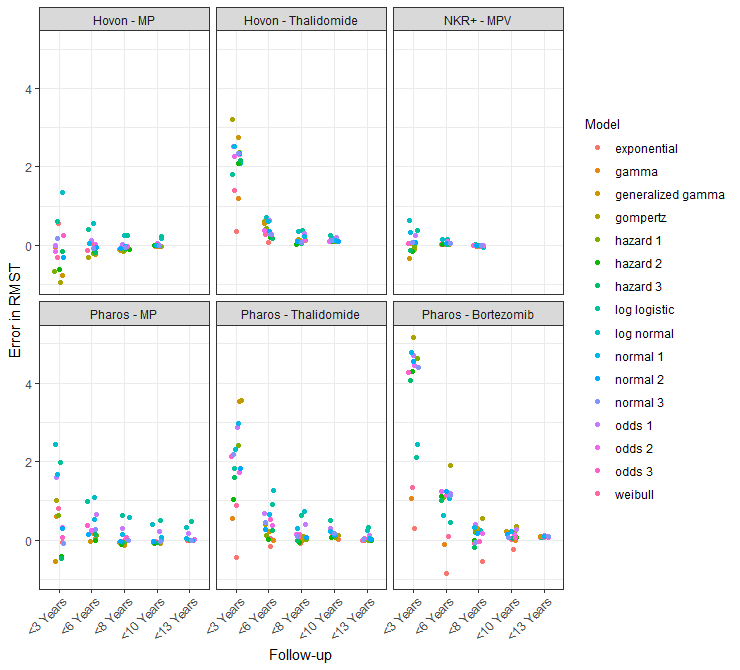


**Figure S13. Absolute Error in RMST according to data source, treatment, follow-up, and model complexity**


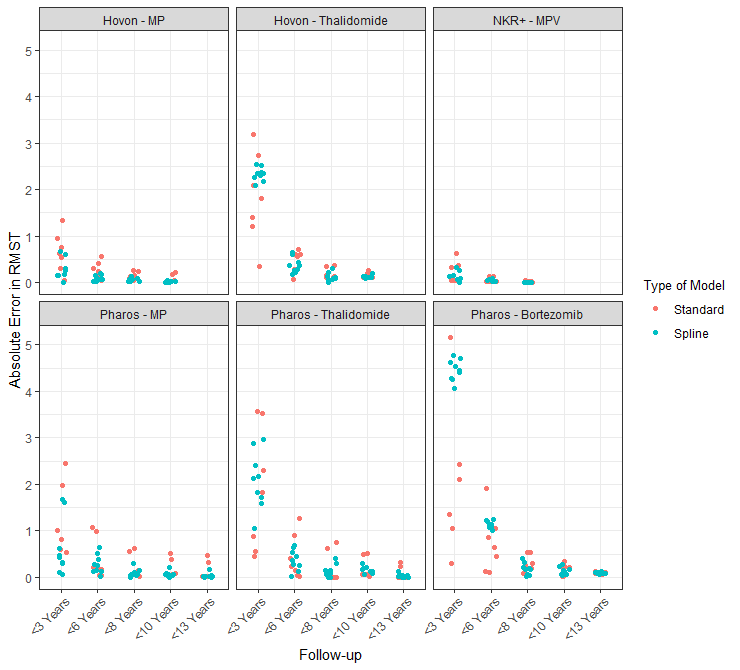


**Figure S14. Error in RMST according to data source, treatment, follow-up, and model complexity**


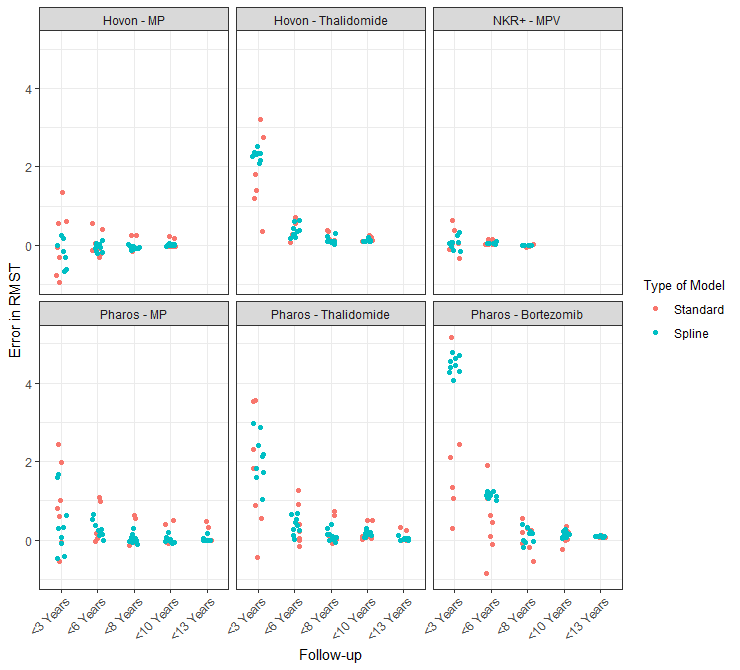


**Figure S15: Absolute RMST error according to the percentage censored and the type of model. The time horizon for RMST in this figure was 8 years for all datasets.**


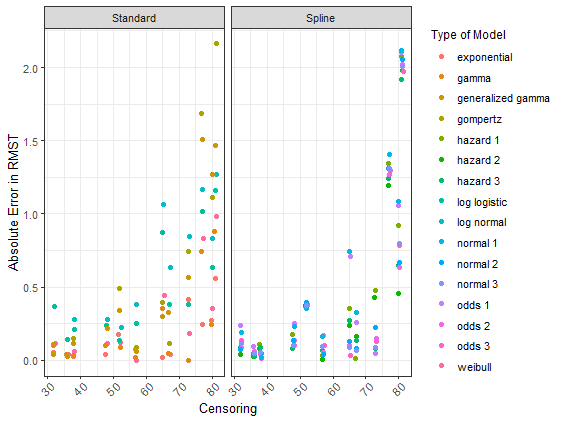


**Figure S16. Absolute Error in RMST according to the absolute number of events and the type of model restricted to 8 years of follow-up.**


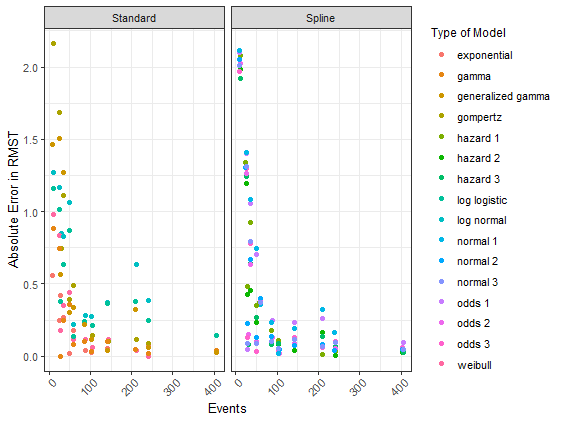


**Figure S17. Error in RMST according to the absolute number of events and the type of model restricted to 8 years of follow-up.**


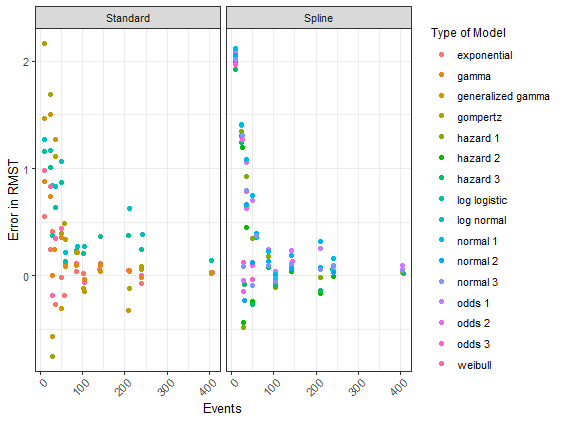


**Figure S18. Error in RMST conditional on survival until extrapolation according to the absolute number of events and the type of model. RMST is estimated for a time horizon of 8 years and a maximum follow-up of 3 and 6 years.**


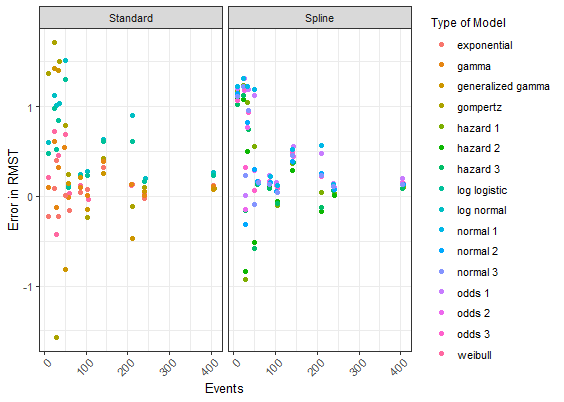


**Figure S19. Error in RMST conditional on survival until extrapolation according to data source, treatment, follow-up, and model. Here the spread for the Pharos data is much larger given that the time horizon is larger (14 years Pharos vs 11 years HOVON vs 8 years NKR+).**


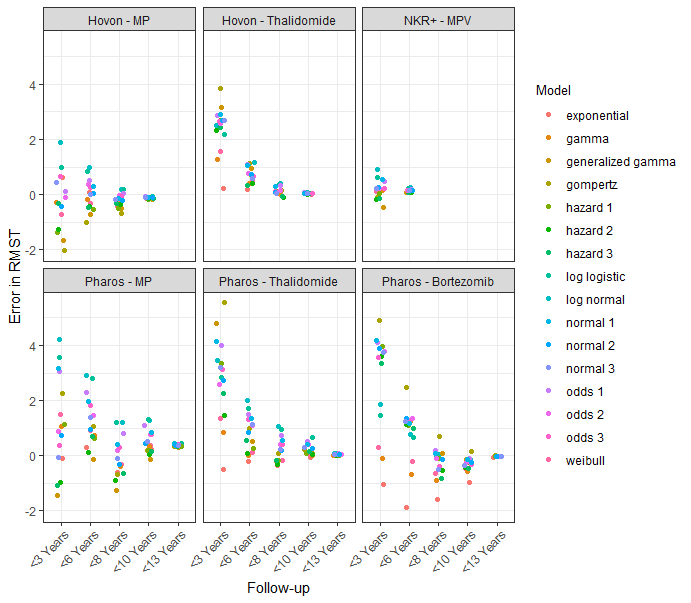

Supplement: Supplementary file 1 — Supplementary Material 1 [file 12874_2023_1952_MOESM1_ESM.docx]
